# Supplementary material for: Manipulating the delivery and immunogenicity of DNA vaccines through the addition of CB[8] to cationic polymers
Source: Mol Ther Nucleic Acids. 2025 Jun 30;36(3):102585. doi: 10.1016/j.omtn.2025.102585 (PMC12447565; doi:10.1016/j.omtn.2025.102585)
Supplement: Document S1. Figures S1–S9 [file mmc1.pdf]

OMTN, Volume *36*

Supplemental information

Manipulating the delivery and immunogenicity of DNA vaccines through the addition of CB[8] to cationic polymers

Hadijatou J. Sallah, Benjamin T. Cheesman, David J. Peeler, Andrew M. Howe, Robin J. Shattock, Roger Coulston, and John S. Tregoning

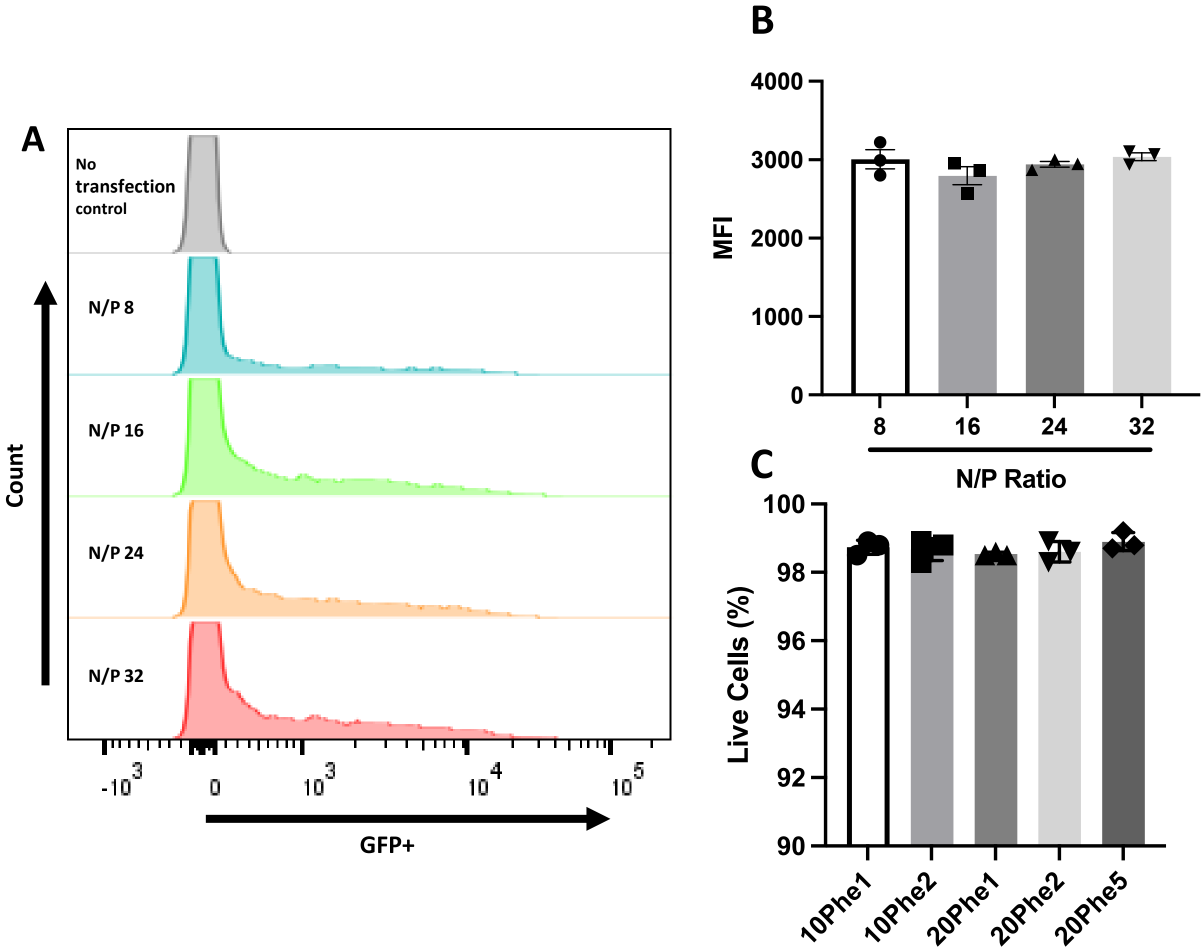

Figure S1. Flow cytometry histograms of live HEK293T following transfection with 20Phe1+CB[8] GFP pDNA formulations at different N/P ratios cells (A), mean fluorescence intensity (MFI) (B) and and cell viability as determined by live/Dead staining (C).

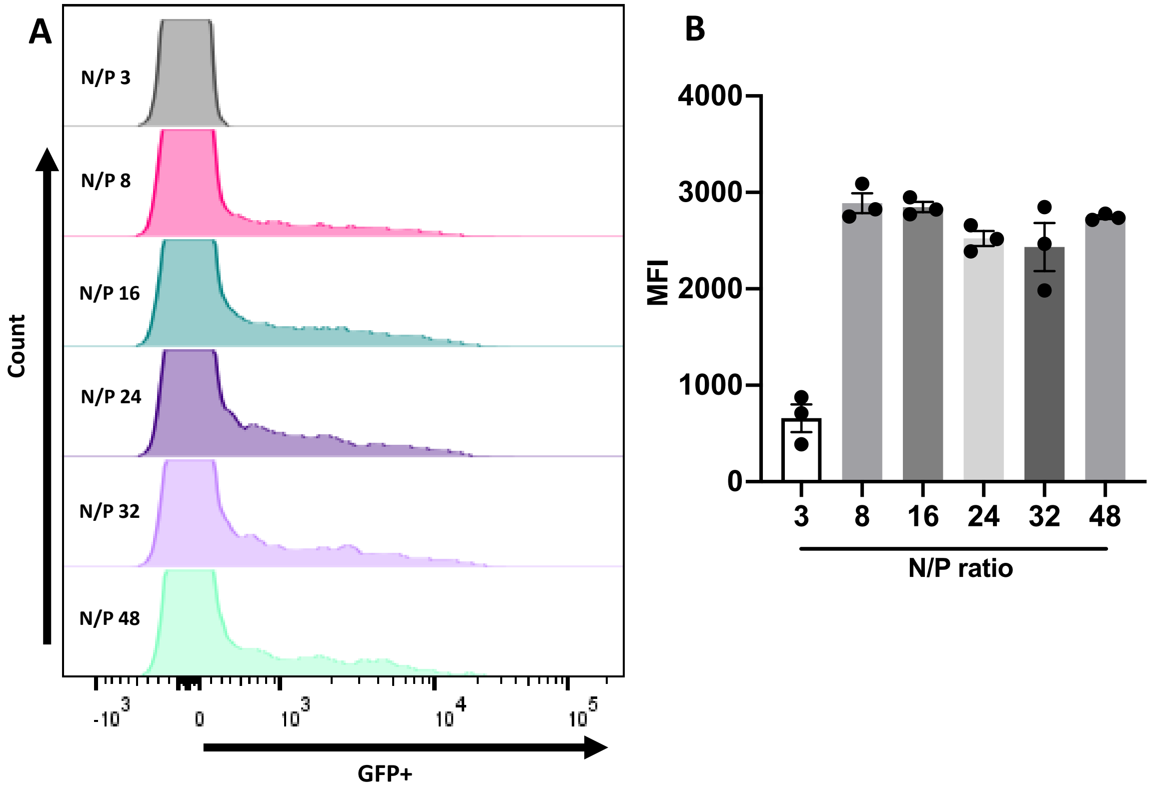

Figure S2. Flow cytometry histograms of live HEK293T following transfection with 20Phe1+CB[8] GFP pDNA formulations at different N/P ratios (A) and mean fluorescence intensity (MFI) (B)

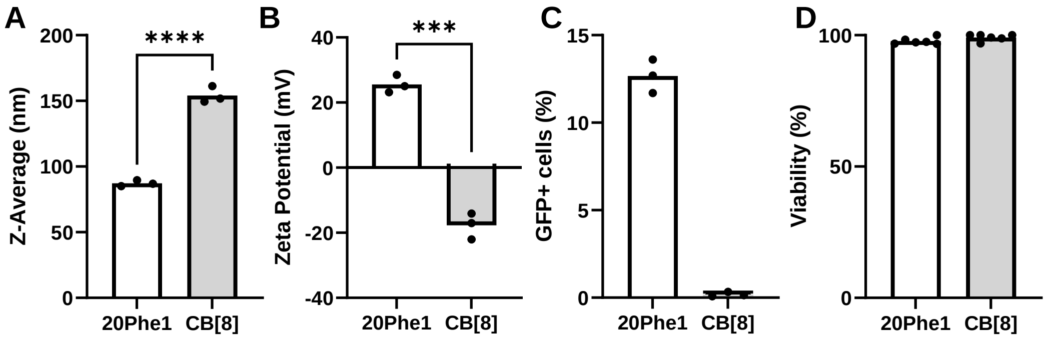

Figure S3. Characterisation of individual polyplex components. Particle diameter (A) and zeta potential (B) of polyplexes formed from CB[8] or 20Phe1 only at N/P ratio of 24:1. Quantification of GFP expression by cell percentage (%) 24 h post-transfection (C). Cell viability of cells following transfection (D).

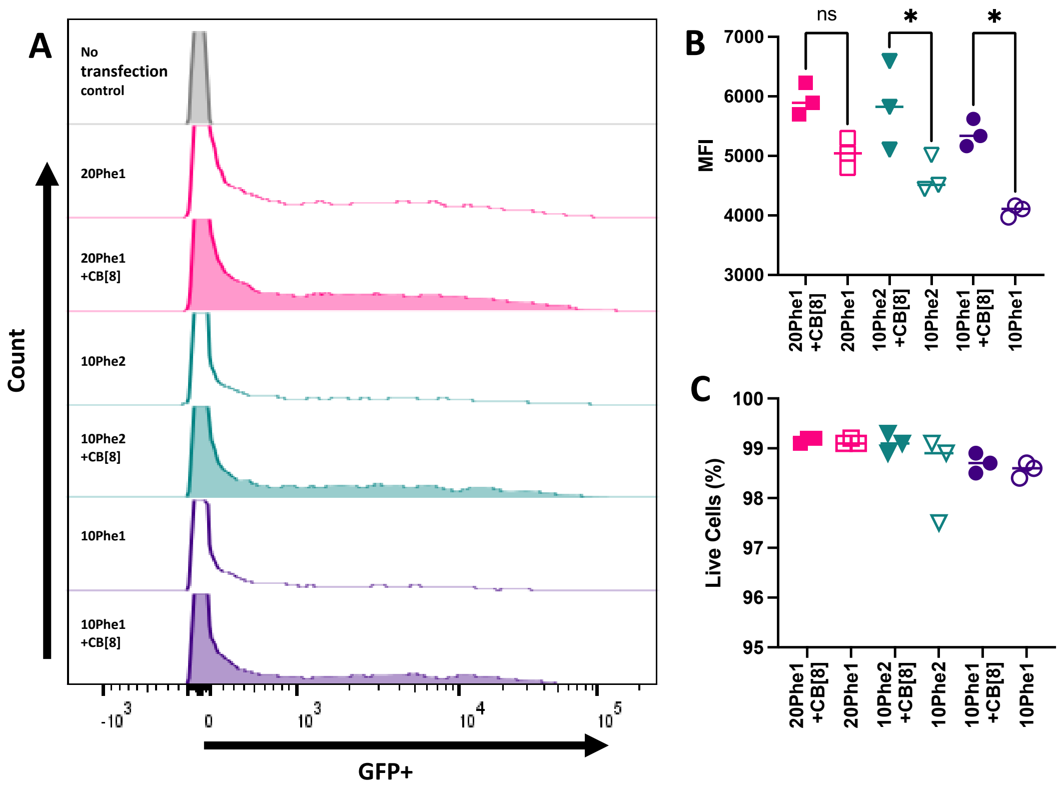

Figure S4. Flow cytometry histograms of live HEK293T following transfection with 20Phe1, 10Phe2 and 10Phe1 ± CB[8] GFP pDNA formulations at different N/P ratios cells (A), mean fluorescence intensity (MFI) (B) and cell viability as determined by live/Dead staining (C) * $p < 0.05$.

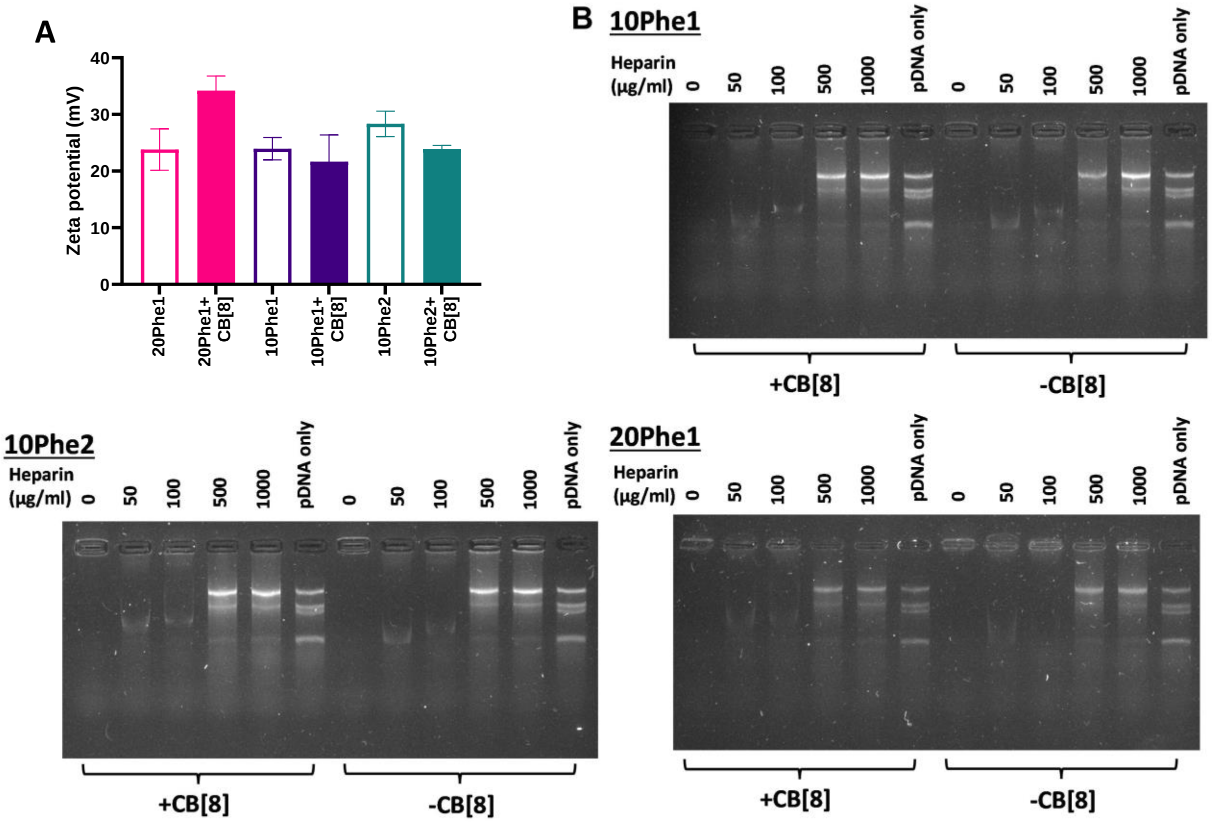

Figure S5. Particle charges and unpackaging of polymer formulations with and without CB[8]. Zeta potential of polyplexes formed using the CB[8] polymer system and pDNA at N/P 32 with or without CB[8] (A).

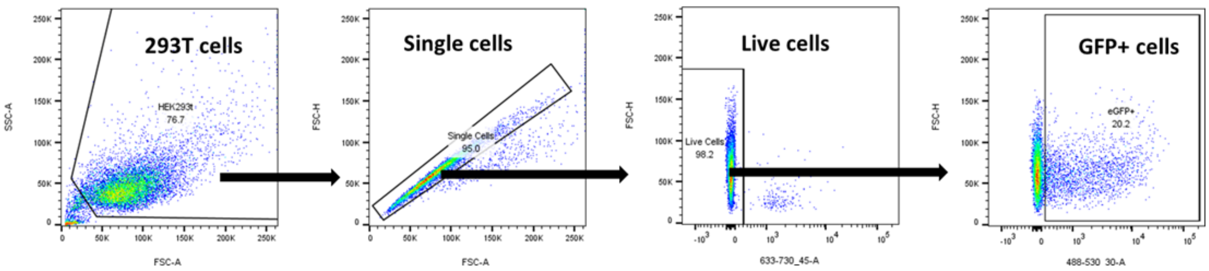

Figure S6. GFP gating strategy. HEK293T/17 cells harvested following transfection and gene expression assessed by flow cytometry assay. Samples were analysed according to gating strategy shown.

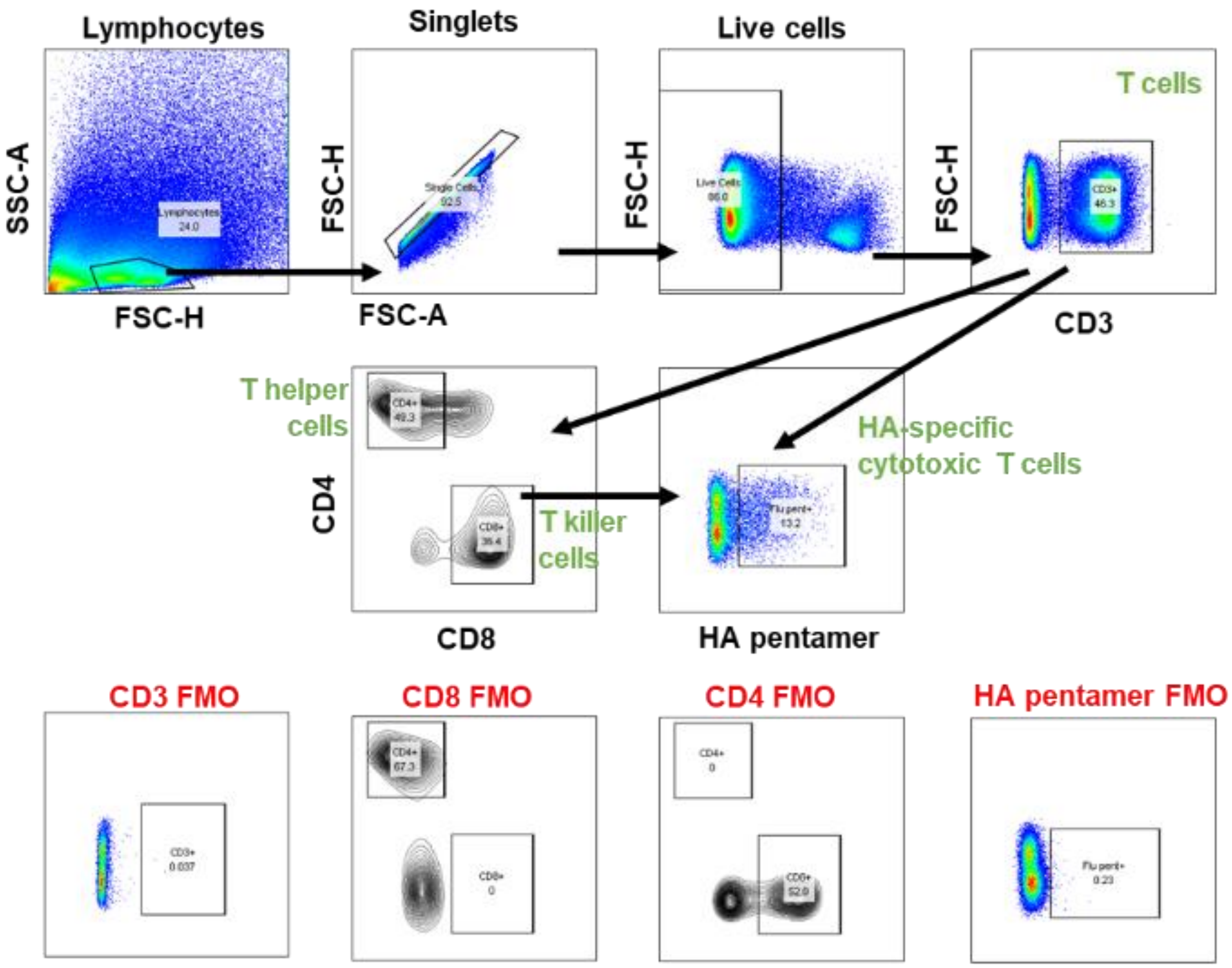

Figure S7. T cell phenotyping gating strategy. Cells were collected from lung prior to flow cytometry assay. Samples were analysed according to gating strategy shown.

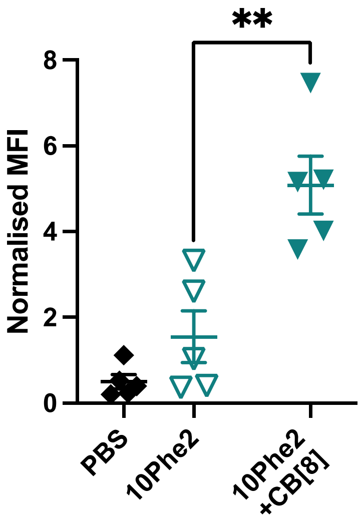

Figure S8. Normalised mean fluorescence intensity (MFI) of live GFP+ cells following administration of 10Phe2 GFP pDNA formulations ± CB[8]. ** $p < 0.005$.

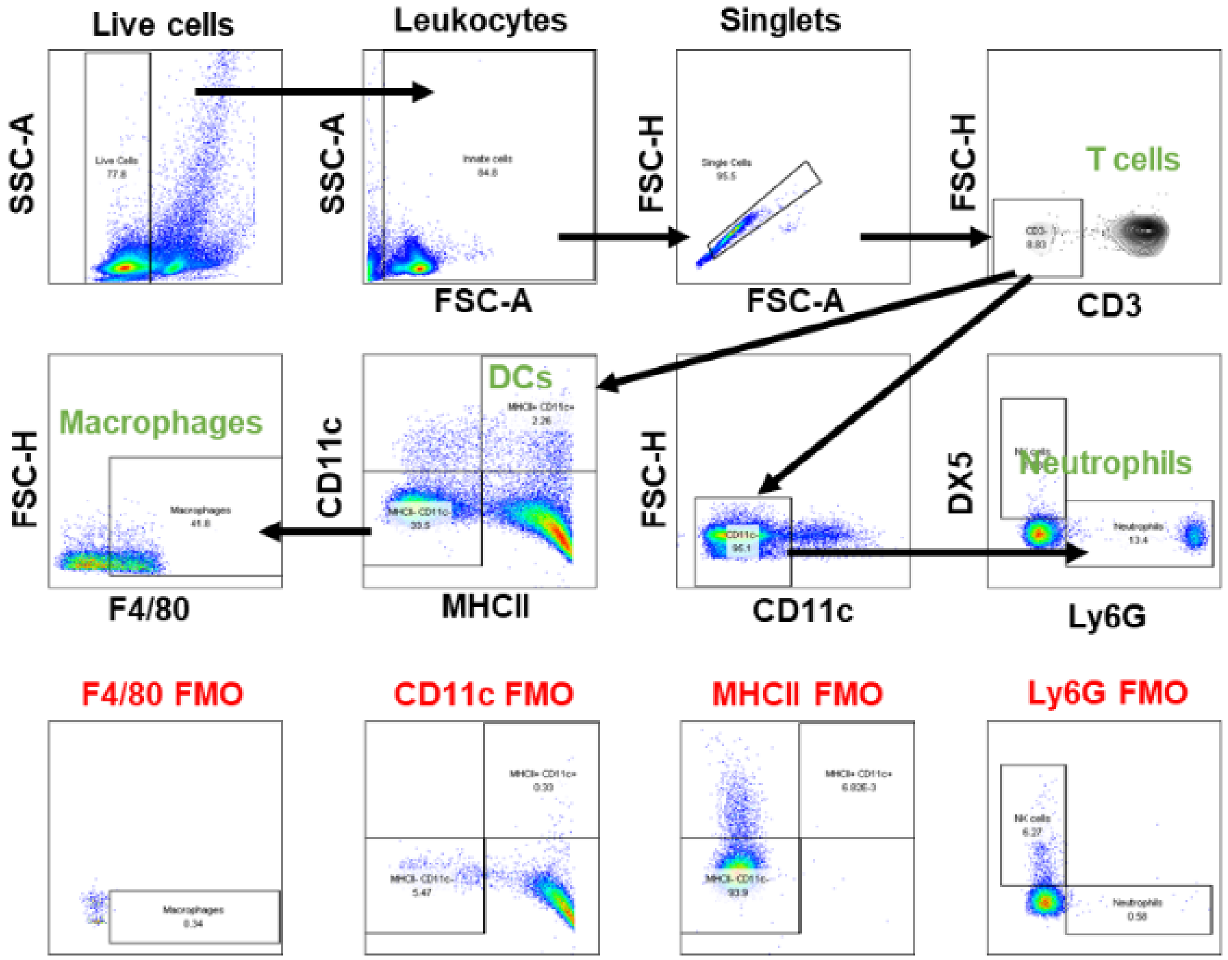

Figure S9. Innate cell recruitment gating strategy. Cells were collected from lymph node or muscle tissue prior to flow cytometry assay. Samples were analysed according to gating strategy shown.
