# Supplementary material for: Manipulating the delivery and immunogenicity of DNA vaccines through the addition of CB[8] to cationic polymers
Source: Mol Ther Nucleic Acids. 2025 Jun 30;36(3):102585. doi: 10.1016/j.omtn.2025.102585 (PMC12447565; doi:10.1016/j.omtn.2025.102585)
Supplement: Document S2. Article plus supplemental information [file mmc2.pdf]

Molecular Therapy
Nucleic Acids
Original Article

Manipulating the delivery and immunogenicity of DNA vaccines through the addition of CB[8] to cationic polymers

Hadijatou J. Sallah,[1] Benjamin T. Cheesman,[2] David J. Peeler,[1,3,4] Andrew M. Howe,[2] Robin J. Shattock,[1] Roger Coulston,[2] and John S. Tregoning[1]

[1]Department of Infectious Disease, Imperial College London, London, UK; [2]Aqdot Ltd, Cambridge, UK; [3]Department of Materials, Imperial College London, London, UK

Challenges with vaccine reactogenicity, stability, and access have highlighted the need to develop alternative strategies for formulation and delivery. We explored the incorporation of cucurbit[n]urils (CBs), as supramolecular "hosts," into nucleic acid-polymer polyplexes. CBs are small, non-toxic, barrel-shaped molecules that transiently crosslink polymers containing supramolecular "guests," thereby increasing molecular weight (MW) of the complex, a correlate of transfection efficiency. We tested whether the supramolecular interactions of CB[8] impact polyplex function. We generated a library of different CB[8] polyplexes using plasmid DNA (pDNA), varying N/P (the ratio of polymer to plasmid), the length, and guest (phenylalanine [Phe]) group frequency of the polyethylenimine (PEI) polymer backbone. We found that N/P 32 and the 20Phe1 (20kDa PEI with 1 mol% Phe) gave optimal gene expression and that incorporating CB[8] in polyplex formulations improved gene expression, both *in vitro* and *in vivo*. Despite increases in gene expression, inclusion of CB[8] in formulations with higher guest-binding capacity led to decreased immunogenicity, possibly as a result of dampened innate immune responses. Our data show that CB[8] polyplexes increase gene delivery and expression but alter inflammatory responses. These findings highlight that rational design of the CB[8] polymer system can enable nucleic acid delivery for both vaccine and therapeutic applications.

INTRODUCTION

Nucleic acid vaccines have been shown to be an effective tool to combat emerging infectious diseases.[1–4] Their efficacy was demonstrated by the successful clinical use of mRNA vaccines in the COVID-19 pandemic.[5,6] Nucleic acids for use as vaccines encode a protein of interest specific to the pathogen they are designed to protect against. Despite the rapid advances in nucleic acid vaccines in the past 5 years, realizing their full potential largely depends on the availability of suitable systems for efficient intracellular delivery. Nucleic acids are negatively charged due to their phosphate backbone, hence uptake of DNA and RNA via endocytosis at the negatively charged cell membrane is very poor.[7] As such, nucleic acids must be formulated to achieve a neutral or positive surface charge, or to increase their lipophilicity through encapsulation, to facilitate cellular uptake.[8] To date, only lipid nanoparticle (LNP)-formulated nucleic acid vaccines have seen widespread clinical use.[5,6] However, LNP formulation raises concerns surrounding reactogenicity, vaccine stability, and intellectual property restrictions, which may reduce equitable access for low- and middle-income countries (LMICs).[9] These issues highlight the need to consider alternative strategies for gene delivery.

An alternative to LNPs is the addition of cationic polymers (polycations) to form complexes with nucleic acid called polyplexes that enable delivery of nucleic acid into cells through glycosaminoglycan binding.[10] Synthetic polycations are attractive candidates for gene delivery as they can be biodegradable, easily synthesized, and stable. Within the polyplex, the nucleic acids are protected from physical and enzymatic degradation and, if the net charge of the polyplex is positive, can be delivered into target cells.[11] PEI is a well-characterized polymer that has been studied extensively for its gene-delivery capabilities. PEI can deliver material into cells with high efficiency; however, while delivery increases with increasing PEI molecular weight (MW), toxicity also increases.[12] Therefore, a challenge is to increase polymer MW without inducing the apparent efficacy-toxicity trade-off.

One method to increase MW-associated transfection efficiency is via supramolecular crosslinking. This crosslinking can be achieved through the addition of cucurbit[n]urils (CB[*n*]s), small barrel-shaped macrocycles composed of glycoluril repeat units bound by methylene bridges.[13] CB[*n*]s have a cavity that is highly hydrophobic and carbonyl-rich portals that are strongly electronegative and capable of hydrogen bonding; thus they interact with "guest" molecules that are hydrophobic, cationic, and/or capable of hydrogen

Received 10 March 2025; accepted 2 June 2025;
https://doi.org/10.1016/j.omtn.2025.102585.

[4]Present address: HS NHLI, Imperial, UK; DP Department of Bioengineering, University of Oregon, USA

Correspondence: John S. Tregoning, Department of Infectious Disease, Imperial College London, London, UK
E-mail: john.tregoning@imperial.ac.uk

© 2025 The Authors.
Published by Elsevier Inc. on behalf of The American Society of Gene and Cell Therapy.
This is an open access article under the CC BY license (http://creativecommons.org/licenses/by/4.0/).

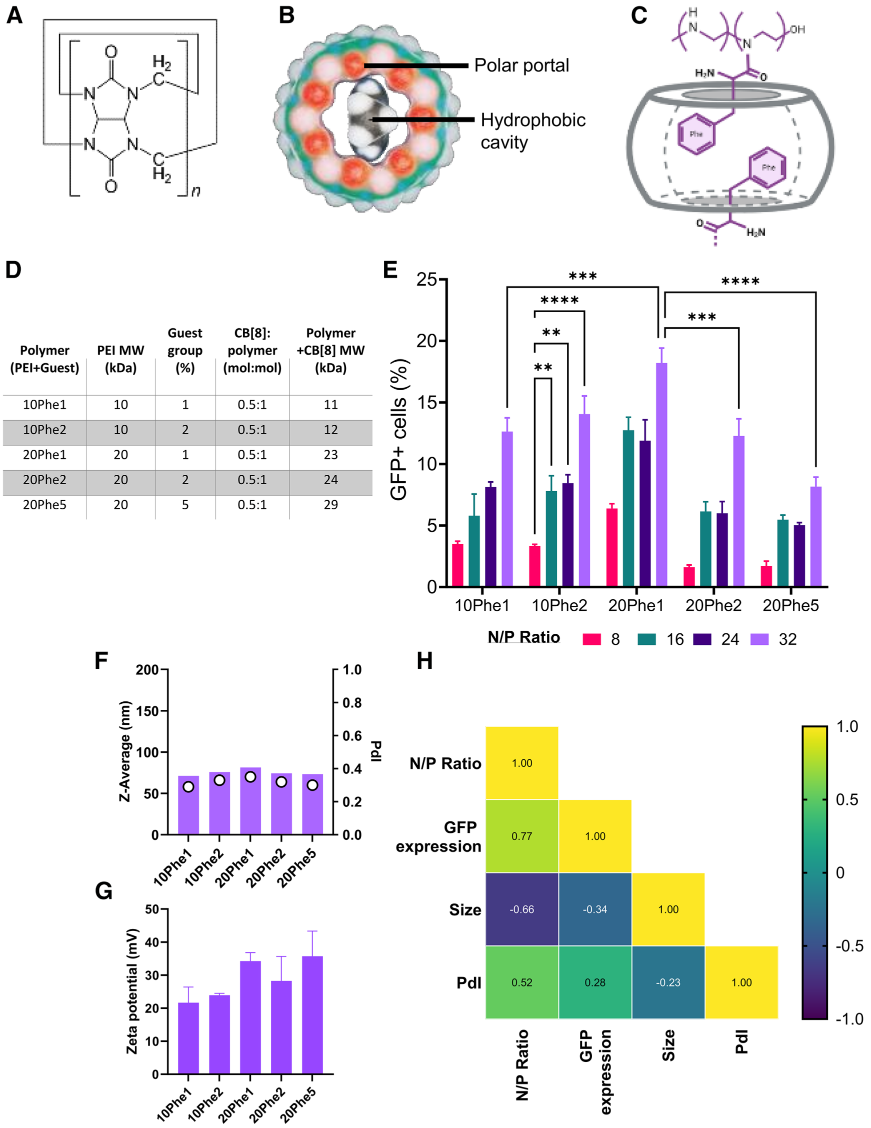

Polymer (PEI+Guest)	PEI MW (kDa)	Guest group (%)	CB[8]: polymer (mol:mol)	Polymer +CB[8] MW (kDa)
10Phe1	10	1	0.5:1	11
10Phe2	10	2	0.5:1	12
20Phe1	20	1	0.5:1	23
20Phe2	20	2	0.5:1	24
20Phe5	20	5	0.5:1	29

Figure 1. Altering polymer size and guest group percentage affects expression level when combined with CB[8]

CB repeat monomer (A), CB[8] structure indicating cavity (B), schematic of interaction between polymers and CB[8] (C). Table indicating variables for design of experiments study (D). Quantification of GFP expression by cell percentage (%) 24 h post-transfection with a library of polyplexes formulated with varying MW, guest-loading capability polymers, and N/P ratios (E). Particle diameter and PdI of polyplexes formed from different CB[8] polymer systems at N/P ratio 32:1 (F). Zeta potential of polyplexes (G). Correlation of characterization data measured against GFP expression (H). Bars represent mean ± SEM; statistical analysis performed by ANOVA with Tukey's test. $^{**}p < 0.01$; $^{***}p < 0.001$.

bonding.[14] Recently, CB[*n*]s have garnered more interest in their biomedical applications, mostly in the form of drug delivery.[15–17] CB[*n*] can interact with rationally designed polymers and reversibly increase MW of the complex through the generation of supramolecular polymer systems. CB[*n*] of different sizes have been suggested as an approach for non-viral gene delivery,[18] for example, CB[7][19] and one study complexing cucurbit[8]uril (CB[8]) with Ru(ii)/tri(bipyridine) to deliver DNA.[20]

In contrast to smaller CB[*n*]s like CB[7] employed by Huang et al.,[19] we utilized (CB[8] for its ability to bind two guest molecules to allow for transient crosslinking of polymer chains. Here, we explored DNA vaccine formulation using supramolecular crosslinking to increase the MW of supramolecular polymer systems by using CB[8] as the host in combination with PEI conjugated to phenylalanine (Phe) guest molecules. Consistent with our hypothesis, we observed that the inclusion of CB[8] increased gene expression *in vitro* and reduced inflammation after immunization. This expression-inflammation profile was associated with a decreased adaptive immune response, indicating a need for a balance between expression and inflammation in the development of nucleic acid vaccine formulations.

RESULTS

CB[8] polyplexes containing 20Phe1 and NP/32 provide optimal expression and immunogenicity

The polyplexes we are exploring have three elements, the plasmid DNA (pDNA) encoding the gene of interest, the cationic polymer that binds the pDNA, and the CB[8] host that crosslinks the polymer. CB subunits (Figure 1A) polymerize to form barrel-like structures with a polar portal and a hydrophobic cavity (Figure 1B). Within the polyplex, each CB[8] molecule binds two Phenylalanine (Phe) guests on the PEI backbone (Figure 1C). Our initial studies explored a range of formulations in the context of CB[8]. We used 2-amino-3-phenylpropanamide-grafted PEIs (Figure 1B; side chain approximates Phe) of varying molecular weights (10 or 20 kDa) and a different-percentage of Phe guest groups (1%–5% per mol); increasing the amount of Phe increases the potential number of CB[8] molecules that can bind to the polyplex as well as its hydrophobicity (Figure 1D). We compared the effect of polymer formulation on GFP pDNA transfection in HEK293T cells across a range of the nitrogen (polymer) to phosphate (nucleotide) ratios (N/P). Increasing polymer molecular weight (MW) from 10 to 20 kDa led to a significant increase in expression (Figure 1E). Increasing the guest loading capacity of the 20 kDa polymers from 1% significantly reduced the GFP expression. Overall, the best expression was seen for 20Phe1 (20 kDa, with

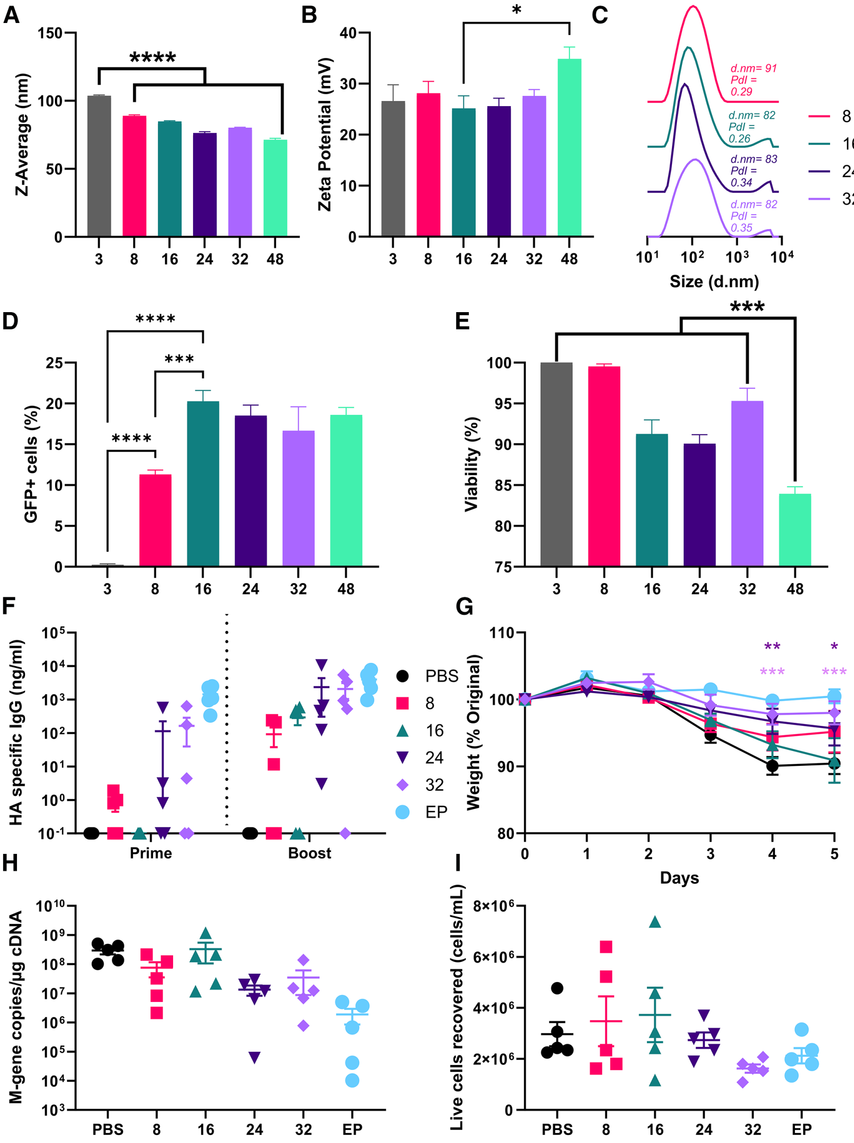

Figure 2. Increasing N/P ratio of CB[8] polyplexes improves transfection efficiency of plasmid DNA *in vitro* and immunogenicity *in vivo*

Particle diameter (A) and zeta potential (B) were assessed of polyplexes formed using the CB[8] polymer system and pDNA at N/P ratios ranging from 3:1 to 48:1. Diameter of 20Phe1 CB[8] polyplexes and different N:P ratios measured by DLS (C). GFP expression was assessed by flow cytometry, presented as % cell, 24 h after transfection (D). Toxicity of polyplexes on cells (E). BALB/c mice were immunized IM with 5 µg of HA pDNA in a prime-boost regime with a 3-week interval between immunizations. HA-specific IgG determined by ELISA from blood collected 3 weeks after both prime and boost (F). Three weeks after immunization, mice were intranasally infected with A/California/07/2009 (H1N1); weight loss (G); viral load (H); and cell recovery (I) after infection. (A, B, D, E) Error bars represent mean ± SEM for $n = 3$. (F–I) $n = 5$; symbols represent individual mice; error bars represent mean ± SEM. Statistical analysis was performed by ANOVA with Tukey's test. $^{**}p < 0.01$; $^{***}p < 0.001$.

1 mol% Phe incorporation). Across different polymers, the sizes ranged from ∼60 to 80 nm with similar polydispersity at ∼0.3 (Figure 1F). There was no difference in zeta potential (Figure 1G). Correlations between particle characterization and expression data were tested to identify the physical characteristics affecting transfection efficiency. In agreement with other polyplex literature,[21] Pearsons's rank correlation indicated that only N/P correlated strongly with expression level, indicating that particle performance was better described by compositional changes not captured by standard physicochemical assays (Figure 1H). Further analysis of gene expression did not show any difference in mean fluorescence intensity (MFI) across these N/P ratios, suggesting that N/P ratio increases incidence of transfected cells but not the amount of gene expressed by each cell (Figures S1A and S1B). There was no difference in cell viability (Figure S1C).

Since N/P most strongly correlated with expression levels, we explored this relationship further. We focused on a 20 kDa PEI polymer with 1% Phe by molecular weight guest moiety (20Phe1) and CB[8] at a molar ratio of 0.5:1 (CB [8]:20Phe1). Under flow-controlled mixing conditions, the combination of 20Phe1+CB[8] and pDNA yielded nanoparticles with a size distribution of approximately 80–100 nm (Figure 2A) and positive surface charge (Figure 2B). Polyplexes formed at N/P ratio of 3 were significantly larger in size than formulations with higher polymer concentrations (Figure 2A); particles at N/P ratio of 48 had significantly higher Zeta potential (Figure 2B). Increasing CB[8] polyplex N/P ratio to 16 and higher resulted in the appearance of micron-scale DLS peaks not seen at N/P 8 and lower, which may be the result of excess CB8-PhePEI aggregating in solution (Figure 2C). HEK293T cells were transfected with GFP pDNA polyplexes and transgene expression measured by flow cytometry. GFP expression was observed at N/P ratios ≥8, and there were significant enhancements in expression levels observed between the N/P 3, 8, and 16. Each increase in polymer concentration roughly resulted in a 2-fold increase in GFP expression, plateauing at the N/P 16 (Figure 2D) with a similar trend observed for fluorescence intensity (Figures S2A and S2B). Further increasing the N/P ratio beyond 16 did not lead to any significant change in transfection efficiency. A small reduction in cell viability was observed as the N/P ratio increased, with a significant reduction in viability at N/P 48 (Figure 2E). We also evaluated the transfection efficiency and cytotoxicity of the individual components of the polyplex, namely 20Phe1 and CB[8]. Cells were transfected

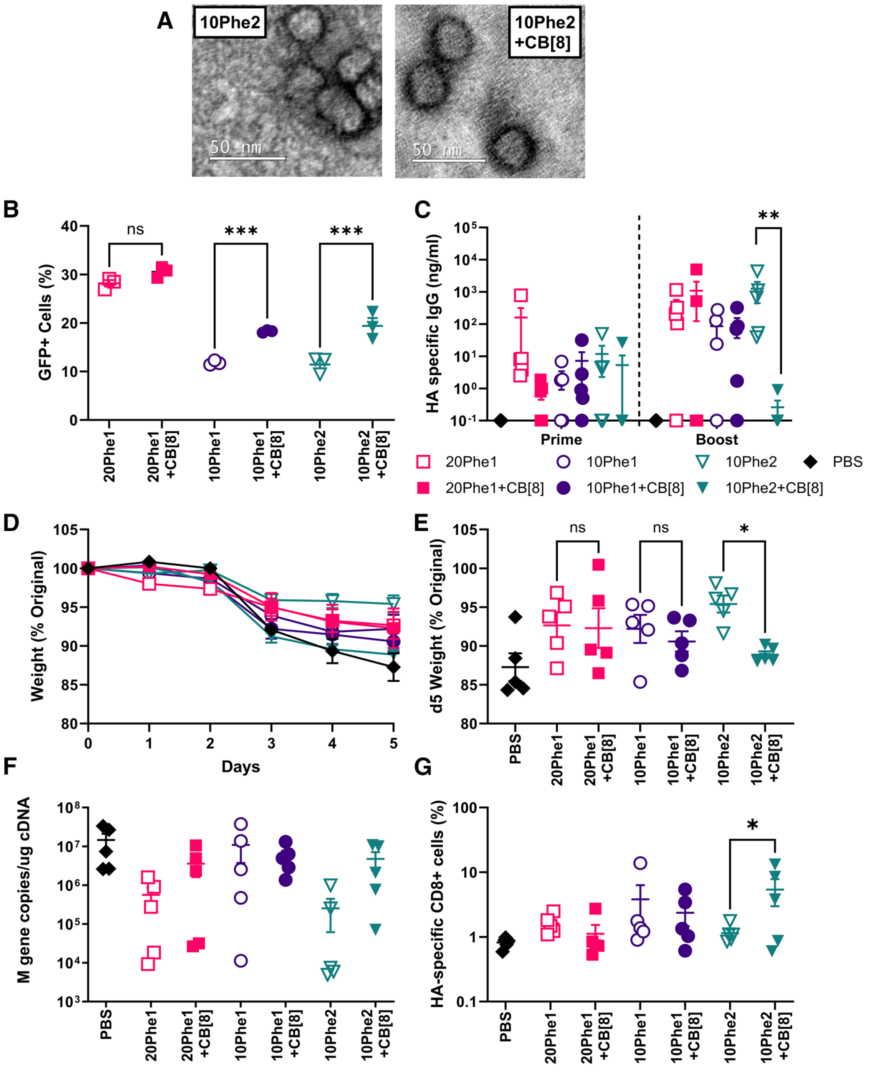

Figure 3. CB[8] increases GFP expression *in vitro* but decreases immunogenicity *in vivo*

TEM of formulated pDNA with and without CB[8] (A). pDNA was formulated at N/P ratio of 32 with various combinations of PEI +/− CB[8], prior to transfection into cells or *in vivo* studies. Quantification of GFP expression by cell percentage (%) 24 h post-transfection of HEK293T (B). BALB/c mice were immunized IM with 5 μg of HA pDNA in a prime-boost regime with a 3-week interval between immunizations. HA-specific IgG determined by ELISA from blood collected 3 weeks after both prime and boost (C). Three weeks after immunization, mice were intranasally infected with A/California/07/2009 (H1N1); weight loss over time course (D) and at d5 (E); viral load (F) and HA-specific CD8 after infection (G). Error bars represent mean SEM for $n = 3$ (B); $n = 5$ (C–G); symbols represent individual mice; error bars represent mean ± SEM. Statistical analysis was performed by ANOVA with Tukey's test. $^{**}p < 0.01$; $^{***}p < 0.001$.

with pDNA formulations at N/P 24, where an equivalent amount of CB[8] was used for the formulation without 20Phe1 (v/v). CB[8]-only (i.e., no polycation) complexes were significantly larger ($p < 0.001$; Figure S3A) and more negatively charged (Figure S3B). Cells transfected with the CB[8]-pDNA-only formulation did not express GFP despite forming nanoscale assemblies with pDNA (Figure S3C) and did not exhibit any toxicity (Figure S3D).

Having seen that altering the N/P ratio in CB[8] polyplexes could alter expression *in vitro*, we then looked at whether they could induce a protective immune response *in vivo* when formulated with pDNA encoding a viral antigen. We compared responses to pDNA-encoded influenza virus hemagglutinin (HA) complexed in CB[8] polyplexes at different N/P ratios. BALB/c mice were immunized intramuscularly (IM) with 5 μg of hyaluronic acid (HA) pDNA in a prime-boost regime with a 3-week interval; DNA delivered by electroporation (EP) was used as a positive control. Anti-HA IgG responses were assessed by ELISA on blood collected 3 weeks after prime or boost immunizations. Antibody responses were low after a single immunization for all conditions except the electroporation positive control, with the highest number of responders in the N/P 24 and 32 groups (Figure 2F). Boosting increased the number of responding animals in all groups, with an increase in titer seen as N/P increased. Mice were subsequently infected with influenza virus A/California/07/2009 (H1N1) 3 weeks post-boost, and weight change was monitored daily as a measure of disease (Figure 2G). Mice in the N/P 24 and 32 polyplex groups lost significantly less weight than the PBS mice on days 4 and 5 after infection; mice immunized with electroporation were protected relative to the PBS from day 3. There was less viral RNA recovered from the lungs of immunized mice compared to PBS controls, with a trend to better protection in the high N/P groups (Figure 2H). There were also fewer cells recruited to the lungs, which is a correlate of inflammation (Figure 2I). These findings suggest that the CB[8] polyplex system is suitable for gene expression *in vivo* and can induce adaptive immunity in mice with N/P 32 giving the greatest protection.

CB[8] increases GFP expression *in vitro* and decreases immunogenicity *in vivo*

Having seen that 20Phe1 complexes at N/P 32 gave the highest expression, we sought to elucidate the impact of CB[8] on these complexes. We first used transmission electron microscopy (TEM) to elucidate the effect of CB[8] on nanoparticle structure, which revealed no obvious morphological difference between particles with and without CB[8] (Figure 3A). We then compared the impact of

inclusion on GFP expression in HEK293T; we compared the effect of CB[8] on three different PEI backbones. The addition of CB[8] significantly increased transfection efficiency for 10 kDa but not 20 kDa PEI polymers (Figure 3B), with a significant increase in MFI for 10 kDa polymers when CB[8] was added (Figures S4A and S4B). Formulations ± CB[8] exhibited minimal reduction in cell viability, with no differences observed between formulation pairs (Figure S4C). We further sought to investigate whether supramolecular crosslinking with CB[8] alters pDNA packaging or unpackaging. There was no difference in polyplex zeta potential ± CB[8], indicating that the excess polycation dominates polyplex surface charge regardless of supramolecular crosslinking (Figure S5A). Similarly, supramolecular crosslinking did not enhance or decrease polyplex resistance to pDNA unpackaging by heparin regardless of polycation molecular weight (Figure S5B). Thus, CB[8] enhances 20Phe1 polyplex transfection through mechanisms unrelated to a modification of polyelectrolyte electrostatic interactions.

Given the increased expression observed following the addition of CB[8], we explored whether these formulations would improve immunogenicity. BALB/c mice were IM immunized two times with 5 μg of HA pDNA prepared at N/P 32 with or without CB[8]. The highest HA-specific antibody titers were observed in the 20Phe1 and 10Phe2 groups; however, the addition of CB[8] to 10Phe2 resulted in a significantly lower immunoglobulin G (IgG) response, an effect that was not seen for 20Phe1 or 10Phe1 (Figure 3C). Three weeks after the boost, mice were challenged with Cal/09 influenza virus. When compared to the naive group, the formulation that provided most significant protection was 10Phe2; however, when CB[8] was added, the protection provided by 10Phe2 was lost (Figures 3D and 3E). This loss was not seen for the other formulations. 10Phe2 alone had the lowest viral load (Figure 3F). Formulation of 10Phe2 with CB[8] resulted in a significantly higher percentage of HA-specific CD8 T cells in the lung when compared to 10Phe2 only (Figure 3G). Overall these data suggest that the addition of CB [8] can dampen immunogenicity elicited from vaccination.

CB[8] dampens innate immune responses *in vitro* and *in vivo*

We next transfected the THP1 human monocyte cell line to investigate the reasons for differential adaptive immune response induced by the inclusion of CB[8]. Cells were transfected with fLuc pDNA formulated in 10Phe2 ± CB[8] at N/P 32. Luciferase activity was not statistically significantly higher in cells transfected with polyplexes containing CB[8] (Figure 4A). Nevertheless, supernatants collected from the transfected cells were used to assess innate immune responses, and 10Phe2 formulated without CB[8] induced significantly higher interferon β (IFN-β), CXCL10, CCL2, and CCL7 (Figure 4B).

Mice were immunized with the same formulations and responses measured in sera 24 h after immunization. There were slightly elevated interleukin-6 (IL-6) and Kupffer cell (KC) levels in the serum of mice immunized with formulations without CB[8] (Figure 5A). In a separate study, we assessed innate cell recruitment

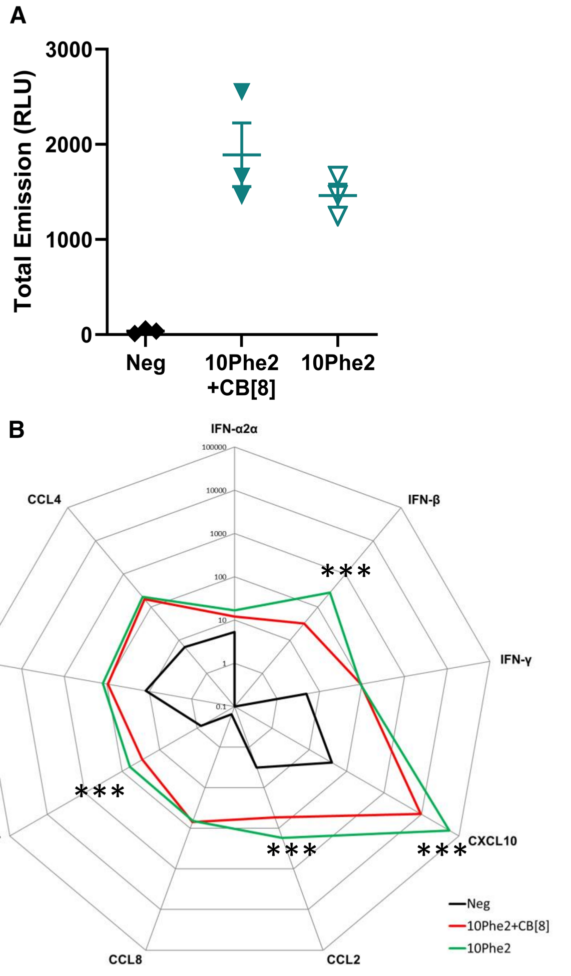

Figure 4. CB[8] dampens innate immune responses in THP-1 cells
Quantification of fLuc expression in relative light units (RLU) in THP1 cells 48 h after transfection with 1 μg of pDNA polyplexes +/− CB[8] (N/P 32) (A). $n = 3$, where symbols represent technical replicates; error bars represent SEM. Cytokine responses assessed by MSD from supernatant of THP1 transfection at 48 h; each point represents mean pg/mL (B). Statistical analysis was performed by ANOVA with Šídák test as a paired comparison between +/− CB[8]. ***$p < 0.001$ between 10Phe2 +/− CB[8].

to muscle and lymph node tissues by flow cytometry 24 h following immunization; in this study, we used GFP pDNA to track transfected cells. In the muscle, there was no difference in the total number of cells between the two polyplex types (Figure 5B), but there were significantly more GFP^+ cells in the group immunized with the CB [8] polyplexes (Figure 5C), and these cells also exhibited increased fluorescence (Figure S8) There were also significantly more neutrophils in the muscle of the group immunized with the CB[8] polyplexes (Figure 5D) but there was no difference in macrophage numbers (Figure 5E). However, the opposite pattern was observed in the lymph nodes. There were significantly more cells in the draining lymph nodes of the group immunized with 10Phe2 without CB [8] (Figure 5F). While there were no differences in neutrophil

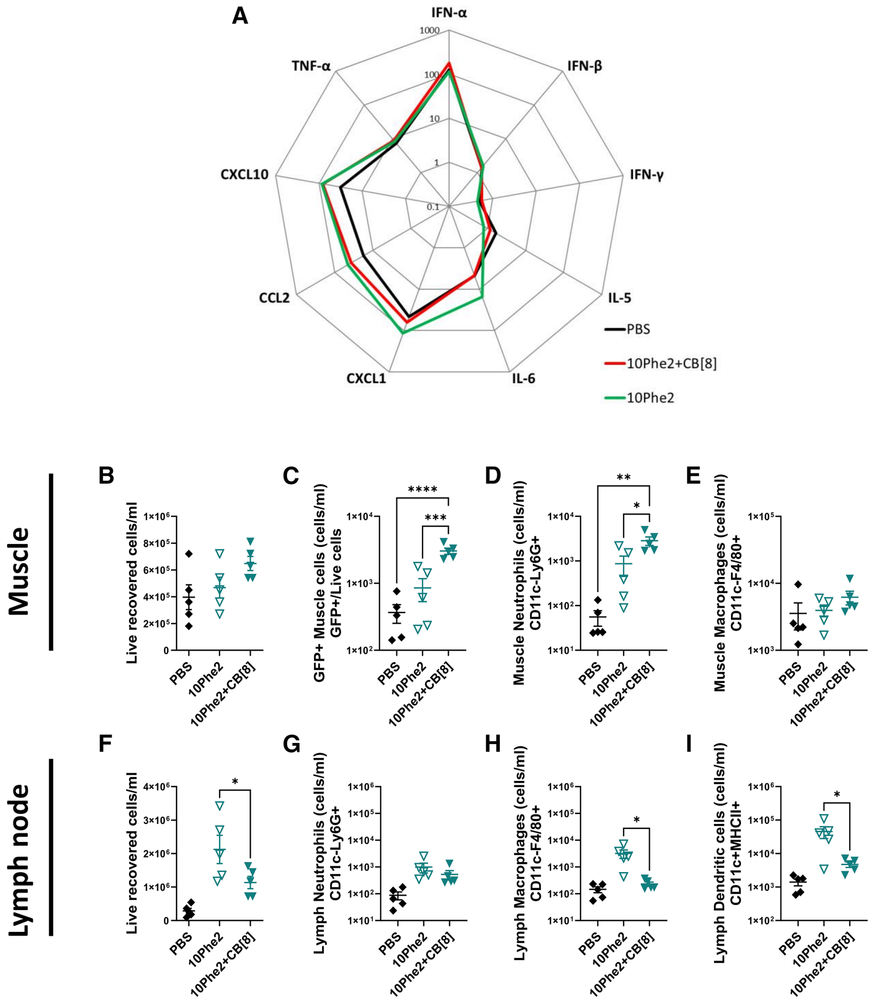

Figure 5. CB[8] increases gene expression *in vivo* but reduces innate cell recruitment

Cytokine responses assessed by MSD from mouse sera 24 h after IM injection with 5 μg of pDNA polyplexes +/− CB[8] (N/P 32) (A); each point represents mean pg/mL. *n* = 5; statistical analysis was performed by ANOVA with Šídák test as a paired comparison between +/− CB[8]. Cell counts of muscle tissue collected 24 h after administration of 20 μg of polyplex formulated GFP pDNA (B). Live muscle cells expressing GFP (C), muscle neutrophils (D), and muscle macrophages (E). Cell counts of lymph nodes collected 24 h after administration of 20 μg of polyplex-formulated GFP pDNA (F); lymph node neutrophils (G); lymph node macrophages (H); and lymph node cDC2 (I). *n* = 5, where symbols represent individual mice; error bars represent SEM. $^{*}p < 0.05$ $^{**}p < 0.01$, $^{***}p < 0.001$, and $^{****}p < 0.0001$ as indicated. Statistical analysis was performed by ANOVA with Šídák test as a paired comparison between +/− CB[8].

numbers in the lymph node (Figure 5G), there were significantly more macrophages (Figure 5H) and cDC2 (Figure 5I) in the 10Phe2 alone group.

DISCUSSION

In this study, we assessed the application of a novel vaccine-delivery platform using cucurbiturils (CB[8]) and polycations as supramolecular hosts and guests, respectively. We observed that when combined with guest-functionalized PEI, CB[8] polyplexes increased transfection efficiency *in vitro* and *in vivo* but diminished adaptive immune response to a pDNA-encoded vaccine antigen. To understand the mechanism of this effect, we investigated innate responses to the formulations, which revealed that formulations with CB[8] dampened cellular and systemic inflammation, suggesting that CB[8] may have a "silencing" effect. Although we did not directly demonstrate the formation of supramolecular structures with CB[8], this has been observed in other studies. Zhang et al. demonstrated condensation of DNA with supramolecular polycations based on interactions between the macrocyclic molecule CB[8] and naphthalene; however, no transfection experiments were performed.[20] These prior studies reveal another key advantage of supramolecular polymers, namely the ability to form broad libraries of copolymers for structure-activity studies simply by mixing solutions of different self-assembling monomers.[22]

Among the wide range of formulation conditions that can affect nucleic acid delivery, we observed that increasing N/P increased transfection efficiency, plateauing at N/P 16. Although increasing the N/P ratio resulted in higher transfection efficiencies, increased N/P ratios have been shown to increase toxicity, which has been associated with higher amounts of free polymer.[23,24] On the other hand, free polymers have also been shown to improve the endocytosis of polyplexes.[23–25] This balance may explain why efficiency plateaus at N/P 16, with toxicity counteracting increased gene delivery. As well as increasing transfection efficiency, increased N/P ratio increased immunogenicity for our polymer system. In addition to N/P ratio, molecular weight and guest loading capacity (the mol% of guest groups) may affect polyplex formation and gene expression.[11,26–28] We tested the effects of these characteristics by generating a library of formulations that assessed both polyplex characteristics and the resulting gene expression. Increasing the guest loading capacity did not change the transfection efficiency for the 20 kDa polymer. Increased polymer MW has been associated with improved transfection,[29] but this is not seen for all polymers.[12,30] As this was a proof-of-concept study in order to determine effects of CB[8], we utilized a 20 kDa PEI, a relatively high MW polymer, that we expected to transfect well and would also induce a measurable level of toxicity. While crosslinked LMW PEIs have been used in literature as a strategy to overcome toxicity, commercial "transfection grade" PEIs are >20 kDa. Additionally, in our study, the use of LMW PEI has the potential to increase shielding of the

polymer, which may have an impact on transfection efficiency. Further optimization of this system can explore strategies to employ LMW PEI such as distancing of guest groups from the PEI backbone to investigate this and assess the application of LMW PEI. Another observation was that increasing the Phe grafting density altered expression; we speculate that this decreases the positive charge density and increases overall hydrophobicity, both of which lead to decreased ability to rapidly release cargo. Decreased charge density could also affect endosomal escape. The need to balance these properties is a known dilemma.[31]

Having identified the optimum MW and N/P ratio, we explored the impact of the inclusion of CB[8] on gene expression and downstream immunogenicity. Nucleic acid vaccines need to be expressed to induce an adaptive immune response, and some studies have observed a relationship between expression and immunogenicity.[32] However, the downstream adaptive immune response to nucleic acid vaccines is multifactorial, and antigen expression is only one component. In our system, the addition of CB[8] improved transfection efficiency *in vivo* but it significantly reduced immunogenicity, particularly the antibody response. Another important component of the response is the inflammation induced by polyplex delivery, which may result from reactions to either polymer, nucleic acid, or the bound polymer-nucleic acid complex.[33] We observed that formulations with CB[8] induce lower cytokine responses *in vitro* and changes to the recruitment of innate immune cells *in vivo*, with increased recruitment to the injection site but reduced recruitment to the lymph nodes. This observation is consistent with CB[8] modulating the inflammatory response to the vaccine, which in turn alters the innate immune cell response and reduces the adaptive immune response. Although the molecular mechanism underpinning this effect is unknown, studies investigating other macrocycles have also observed immunomodulatory effects. Use of the adjuvant AS01 altered neutrophil antigen trafficking to lymph nodes.[34] A study found that hydroxypropyl-β-cyclodextrin (BCD) molecules lead to a significant reduction in pro-inflammatory cytokines from peripheral blood mononuclear cells (PBMCs) that had been exposed to cholesterol crystals, which is known to induce inflammation.[35] Another study assessing immunosafety of CBs in BALB/c mice found that high concentrations of CBs affected the immune system, with different CB homologues changing the cellular immune profile and CB[6] appeared to stimulate more B cells than CB[7] and CB[8] but showed reduced immune response stimulation with SRBC antigen.[36]

In this study, we evaluated the applicability of CB[8] polyplexes as vaccines, which revealed an unexpected dampening of innate inflammation. Despite enhanced gene expression, CB[8] polyplexes were not sufficient to provide humoral protection following viral challenge. However, for other approaches, such as protein replacement, increased expression with reduced inflammation may be beneficial. This effect highlights the important role, among many, that formulation plays in subsequent vaccine response beyond transfection efficiency. Our findings show that the CB[8] polyplex system is a novel and viable delivery system for nucleic acids, and its "silencing" capabilities provide the opportunity for potential therapeutic applications beyond the reach of inflammatory delivery systems.

MATERIALS AND METHODS

Polyplex components

CB[8] ($C_{48}H_{48}N_{32}O_{16}$) was also supplied by Aqdot. A range of linear PEI polymers conjugated to Phe was synthesized by Aqdot (Cambridge, UK), which includes 10Phe1, 10Phe2, 20Phe1, 20Phe2, and 20Phe5. All polymers have been named according to PEI molecular weight (MW/kDa) and Phe percentage (moles Phe to moles PEI). For example, 20Phe1 describes a 20 kDa PEI with 1 mol% of the PEI monomers on average having an attached Phe or 4–5 Phe per 20 kDa EI chain. Below is an example of 20Phe1 synthesis. Other molecular weights and Phe loadings were prepared by varying the linear PEI starting material and reagent loadings accordingly.

Neutral linear PEI was obtained by dissolving linear PEI hydrochloride salt (2.00 g, 20 kDa, Sigma Aldrich) in water (12 mL) followed by addition of triethylamine (0.4 eq., 1.04 g). A white precipitate formed upon neutralization, which redissolved upon heating at ∼80°C. Solvent was then exchanged by adding DMSO (24 mL) and evaporating the water on a rotary evaporator. The resulting clear very pale-yellow solution was cooled to room temperature, then Boc-Phe-OSu in DMSO (0.037 eq., 0.339 g in 8 mL DMSO) was added dropwise by syringe. After complete addition, the reaction mixture was heated at 80°C. After ∼19 h of reaction, the cloudy yellow solution was cooled to room temperature and 5M HCl (20 mL) was added, causing to form an off-white precipitate. Sufficient water was added to dissolve the precipitate. The clear very pale-yellow solution was dialyzed against water (3.5 kDa MWCO dialysis membrane) and freeze dried to recover elastomeric yellow solid product (1.413 g). The degree of Phe guest loading was estimated by 1H NMR, as guest loading measurement by UV was not possible due to PEI absorbance in the region of interest. The chlorine and nitrogen content were determined by elemental analysis. The N:Cl ratio of the product was adjusted to 1:1 give a final material which can be used directly after dissolution in water by dissolving a known mass in water (∼5 mL), addition of HCl (5M, 15.49 g), and addition of further water (20 mL) to dissolve the white precipitate formed. The clear yellow solution was freeze-dried to yield an off-white powder product (3.681 g). The guest loading was re-checked by 1H NMR, and an N:Cl ratio of 0.96:1 was measured by elemental analysis.

Cell culture

The cell lines utilized in this study were HEK 293T/17 (ATCC, US) and THP-1 (ATCC, US). HEK293T/17 cells were cultured in complete DMEM (cDMEM) (Thermo Fisher, UK) containing 10% (v/v) fetal calf serum (FCS), 1% l-glutamine, and 1% penicillin/streptomycin (Thermo Fisher, UK). THP-1 cells were cultured in complete RPMI-1640 medium (Sigma, UK) containing 10% (v/v) FCS, 1% l-glutamine, and 1% penicillin/streptomycin (Thermo Fisher, UK). All cells were maintained in a humidified incubator at 37°C and 5% CO_2 atmosphere.

Plasmid purification

Transformed *Escherichia coli* for producing plasmid DNA (pDNA) encoding either GFP or haemagglutinin (HA) from the H1N1 A/California/07/2009 influenza strain were cultured in 100 mL of Luria broth (LB) supplemented with 100 μg/mL carbenicillin (Sigma-Aldrich, UK). Isolation of pDNA was performed using a Plasmid Plus Maxiprep kit (QIAGEN, UK). pDNA concentration and purity were determined using a NanoDrop One (Thermo Fisher, UK) spectrophotometer.

Polyplex preparation

Stock solutions of polymers, CB[8], and pDNA were prepared first by directly dissolving these materials in 0.22 μm filtered, molecular grade water. Polymers and pDNA were stored at 4°C for 1 and 3 months, respectively. CB[8] was prepared fresh for every experiment and sonicated to ensure homogeneity of the solution. The concentrations of the stock solutions were 1 and 2 mg/mL (lPEI-Phe), 0.5 and 1 mg/mL (CB[8]), 2 mg/mL (PEI MAX (Polysciences Europe, Germany), and 1 mg/mL (pDNA). PEI MAX was used as a positive control. pDNA stock solution was diluted to a concentration of 60μg/mL using 5% glucose in 0.22 μm filtered molecular grade water (solution A). Predetermined amounts of polymer and CB[8] stock solutions were complexed and each diluted to 1,000 μL using the same buffer (solution B). All solutions were vortexed to ensure homogeneity. Polyplexes were prepared by loading solutions A and B into separate Luer-Lok syringes and infusion at a 1:1 (v/v) ratio using the Standard Infuse/Withdraw PHD ULTRA syringe pump (Harvard Apparatus) with a T-bar junction mixer at a rate of 1 mL/min. The mass of pDNA in each sample was kept constant to compare the effect of the nitrogen (polymer) to phosphate (nucleotide) ratios (N/P), polymer MW, and CB[8] had on transfection efficiency, protein expression, and immunogenicity. A series of PEI-Phe polyplex solutions was prepared with N/P ratios ranging from 3:1 to 48:1.

Dynamic light scattering

Mean diameters (Z-average, D_z) and polydispersity of all polyplexes formed between PEI w/Phe, CB[8], and pDNA in aqueous 5% glucose were determined by dynamic light scattering measured using a Zetasizer Nano ZS instrument (Malvern Instruments, UK). Data were processed using analysis of the correlation of variation and the Stokes-Einstein equation. All measurements were taken at a fixed scattering angle of 173° at 20°C. Formulations were analyzed using folded capillary cuvettes (DTS1070, Malvern Panalytical, UK). Measurements were made with folded capillary cuvettes and formulations loaded using a diffusion-barrier technique.[37]

Zeta potential

Zeta potential measurements were conducted at 25°C using a Zetasizer Ultra (Malvern Instruments) instrument. Formulations were measured at pDNA concentrations of 3 μg/mL in 1 mM NaCl. Data were processed using the Smoluchowski model. Measurements were made with folded capillary cuvettes (DTS1070, Malvern Panalytical, UK)

In vitro transfection of GFP

HEK293T/17 cells were seeded at a density of 7.5×10^5 cells per well in a clear 12-well plate 24 h before transfection in cDMEM. For the transfection, the medium was completely removed and replaced with 1,000 μL of prewarmed transfection medium (DMEM with 1% l-glutamine); 33.3 μL (equivalent to 1 μg pDNA) of the polyplex solution was added to each well and allowed to incubate for 4 h; then the transfection medium was completely removed and replaced with 1,000 μL of cDMEM. After 24 h, cells were harvested and processed for flow cytometry analysis (Figure S6).

Polyplex packaging assay

Polyplexes were prepared with GFP pDNA to N/P 32 in 5% glucose in water and then treated with heparin sulfate (Sigma, UK) at varying concentrations and incubated at 37°C for 30 min. Samples were then loaded into a 1% agarose gel containing TAE buffer and 1:10,000 SYBR Safe Gel Stain (Invitrogen, UK). The gel was run at 130 V for 40 min and imaged using a UV transilluminator (UVP).

Flow cytometry

Cells were detached from plates using 500 μL trypsin (TrypLE Express 1X) (Gibco, UK) and incubated at 37°C for 30 min and quenched using 500 μL of cDMEM. The cell suspensions were then transferred to a 96-well 2.2 mL deep-well plate and centrifuged at 1750 rpm for 7 min. Cells were stained with 100 μL Fixable Zombie NIR Live/Dead Cell stain (BioLegend, UK) dilution 1:400 in PBS for 30 min at RT. Cells were then washed with 2 mL of FACS buffer (PBS +2.5% FCS), centrifuged at 1,750 rpm for 7 min and resuspended and fixed in 250 μL of PBS and 250 μL of 3.0% paraformaldehyde (Thermo Fisher, UK), resulting in a final concentration of 1.5%, washed and kept refrigerated at 4°C until analysis. Samples were analyzed using an LSRII (BD Biosciences, UK) flow cytometer with FACSDiva software (BD Biosciences, UK). The phenotypic identity of GFP-positive cells was quantified using FlowJo version 10.6.2 (FlowJo LLC, USA).

Cytotoxicity assay

HEK293T/17 cells (ATCC, USA) were cultured in cDMEM (Thermo Fisher, UK) containing 10% (v/v) fetal calf serum (FCS), 1% l-glutamine, and 1% penicillin/streptomycin (Thermo Fisher, UK) and maintained in a humidified incubator at 37°C and 5% CO_2 atmosphere. Cells were plated at a density of 50,000 cells per well in a clear 96-well plate 24 h before transfection. For the transfection, the medium was completely removed and replaced with 100 μL of prewarmed transfection medium (DMEM with 1% l-glutamine); 3.3 μL (equivalent to 100 ng pDNA) of the polyplex solution was added to each well and allowed to incubate for 4 h; then the transfection medium was completely removed and replaced with 100 μL of cDMEM. Following incubation, 100 μL of CellTiter-Glo2.0 (Promega, USA) reagent was applied according to the manufacturer's instructions. Absorbance was then analyzed on a FLUOstar Omega plate reader (BMG LABTECH, UK) and readings normalized to a cDMEM-only control.

Transmission electron microscopy

Polyplexes were prepared with GFP pDNA to N/P 32 in water. Ten microliters of preparations were applied directly onto a 300-mesh copper grids with a holey carbon film (Agar Scientific, UK). Samples were stained using 2% (w/w) uranyl acetate, rinsed twice with deionized water, and air-dried. Imaging was performed using a TEM-2100 Plus electron microscope (JEOL USA, Peabody, MA, USA) operated at 80 kV.

In vivo immunization studies

In vivo immunizations were conducted on 6- to 8-week-old, female BALB/c mice (Charles River, Highbury, UK). Mice were housed in groups (n = 5) in fully ventilated cages in an acclimatized room with food and water supplied ad libitum. Mice were immunized using a prime-boost regimen, receiving two doses, 3 weeks apart. Doses were administered via IM injection with 5 μg of HA pDNA complexed with formulations of 10Phe1, 10Phe2, or 20Phe1 with or without CB[8] in 50μL volumes. Naive mice received 50 μL PBS.

All work was approved by the Animal Welfare and Ethical Review Board at Imperial under PPL P4EE85DED. Mice were maintained in autoclaved individually ventilated cages (IVC) under positive pressure, with a mixture of Tapvei Eco-Pure Premium Aspen chips (Datesand) and Sizzle-Pet (1034015; LBS, UK) for bedding. Mice were housed in groups of up to five animals per cage. Mice had *ad libitum* access to irradiated RM3 pellets for food.

Challenge

For challenge studies, mice were challenged with 2.5 × 10^5 pfu of H1N1 A/California/07/2009 influenza virus resuspended in 100 μL of PBS. Virus solution was placed onto their noses, which was then inhaled. This was performed on unconscious mice after being anesthetized with vaporized isoflurane. Mice were weighed at the time of challenge and each day thereafter. Mice were regularly monitored and were culled via overdose, by intraperitoneal administration of pentobarbital to ensure they did not surpass the severity limit of the experiment and cull-confirmed by terminal bleed.

Tissue dissection and sample collection

Blood samples were collected 3 weeks after each dose was administered and serum was extracted and stored at −80°C for ELISA analysis. The right lung lobe was harvested and stored at −80°C for qPCR analysis. The left lung lobe was harvested and processed immediately for flow cytometry analysis. Briefly, lungs were manually homogenized by mashing then passed through 100 μm cell strainers. Lung cells were then centrifuged at 500 xg for 5 min and supernatants removed before treatment with red blood cell lysis buffer (ACK; 0.15 M ammonium chloride, 1 M potassium hydrogen carbonate, and 0.01 mM EDTA, pH 7.2) (Gibco, UK). To remove lysis buffer, cells were washed with RPMI-1640 and centrifuged at 200 xg for 5 min before final resuspension in complete RPMI-1640. Cell concentrations and viability were determined by trypan blue exclusion.

Anti-HA-specific IgG ELISA

A semiquantitative immunoglobulin ELISA was performed by coating 96-well ELISA microplates (Nunc, Rochester, NY) with 1 μg/mL Cal/09 influenza A capture antigen (Sino Biological) and anti-mouse Kappa (1:1,000) and lambda (1:1,000) capture antibodies (for standards) (Novus Biologicals), all diluted in PBS then stored at 4°C overnight. Plates were washed with PBS and Tween 20 and blocked with assay buffer (1% [w/v] bovine serum albumin [BSA] and 0.05% and [v/v] Tween 20 in PBS) for 1 h at 37°C and washed. A series of dilutions of serum samples and 5-fold serial dilutions of purified IgG standard starting at 1,000 ng/mL were added to plates and incubated at 37°C for a further 2 h and washed. Bound antibody was detected by addition of a 1:5,000 dilution of anti-mouse IgG-HRP and incubated at 37°C for 1 h and washed. Plates were developed with tetramethylbenzidine (TMB), and the reaction stopped after 5 min with H_2SO_4. Absorbance was measured using the VersaMax spectrophotometer (Molecular Devices) and SoftMax Pro GxP v5 software.

RT-qPCR

Lung tissue was homogenized in Trizol using the TissueLyser (Qiagen, Manchester, UK) instrument at 50 oscillation for 4 min, then RNA was extracted from homogenates using Trizol/chloroform extraction. Briefly, homogenates were transferred to 1.5 mL tubes, and 150 μL TRIzol reagent was added, followed by a 5-min RT incubation. One hundred microliters of chloroform was then added, and the tubes were inverted to mix. After a 15 min centrifugation at 13,000 xg at 4°C, the aqueous phase was transferred to fresh tubes. RNA was precipitated with 250 μL isopropanol (10-min RT incubation and 10-min centrifugation at 13,000 xg), washed with 75% ethanol (5 min at 7,500 xg), air-dried for 15 min, and resuspended in nuclease-free H2O. Samples were incubated at 55°C for 5 min to denature RNA secondary structures and then placed on ice. Total RNA concentrations and purity were determined using a NanoDrop One (Thermo Fisher, UK) spectrophotometer. All RNA samples were normalized to 200 ng/μL and converted into cDNA using a GoScript reverse transcription system (Promega, UK). RT-qPCR was performed for the influenza M gene on a Stratagene Mx3005p (Agilent technologies, Santa Clara, CA, USA) instrument, using primers 5′-AAGACAAGACCAATYCTGTCACCTCT-3′ and 5′-TCTACGY TGCAGTCCYCGCT-3′, and probe 5′-FAM-TYACGCTCACCGT GCCCAGTG-TAMRA-3. M-gene-specific RNA copy number was determined against an influenza M gene standard.

T cell phenotyping

Lung cells were plated in a U-bottom 96-well plate and centrifuged at 500 g for 2 min at 4°C. Cells were stained with 100 μL of Live/Dead violet dye (BioLegend, Catalog: 423113) and incubated for 20 min at 4°C in the dark. After centrifuging at 500 g for 2 min, the supernatant was removed. Cells were resuspended in Fc block (Anti-CD16, Clone:93, BD, Catalog: 6266549) in PBS with 1% BSA for 20 min, followed by staining with surface antibodies: CD3 (Clone: 17A2, BioLegend Catalog: 100204), CD4 (Clone: RM4-5, BD, Catalog: Invitrogen 45-0042-82), CD8a (Clone: 53–6.7, BD, Catalog: 560182), major histocompatibility complex (MHC) class I Pentamer

(H-2Kd–IYSTVASSL, Proimmune, F240-2A-G) for 1 h at room temperature in the dark. Excess antibodies were washed off three times with 1% BSA in PBS, and the cells were filtered through fluorescence-activated cell sorting tubes. Flow cytometry was performed on an LSR Fortessa (BD), and data analysis was conducted using FlowJo. Fluorescence minus one (FMO) controls were used for surface-staining analysis (Figure S7).

In vitro transfection of firefly luciferase

THP-1 cells were seeded at a density of 10^6 cells per well in a clear 96-well plate 24 h before transfection. For the transfection, the medium was completely removed and replaced with 200 μL of prewarmed transfection medium (DMEM with 1% l-glutamine); 100 ng of fLuc pDNA polyplex formulations was added to each well and allowed to incubate for 4 h; then the transfection medium was completely removed and replaced with 200 μL of complete RPMI-1640. After 24 h, cells were centrifuged for 2 min at 500 g and 100 μL of supernatant removed and stored at −80°C for cytokine analysis. Remaining transfection samples were assessed by luciferase assay. To assess transfection efficiency of fLuc polyplexes in THP-1 cells, 100 μL Bright-Glo luciferin substrate (Promega, UK) was added to wells. After 5 min, 200 μL of the treated samples was transferred to white 96-well plates (Falcon, US), and luminescence intensity was analyzed using a FLUOstar Omega plate reader (BMG LABTECH, UK).

In vivo innate cell recruitment and gene expression study

Mice were injected via IM with a single dose of 5 mg GFP pDNA complexed with formulations of 10Phe2 with or without CB[8] in 50 μL volumes. Naive mice received 50 μL PBS. Mice were culled 24 h post-injection. Blood samples were collected and serum extracted and stored at −80°C for mean squared displacement (MSD) analysis. Lymph nodes were removed and digested in DNase (200 μg/mL) for 15 min before being homogenized as described earlier. Injection site muscles were harvested and digested in DNase (200 μg/mL), Liberase (12.5 μg/mL), and Hyaluronidase (50μg/mL) for 15 min before being homogenized and processed as described earlier.

In vivo innate cell recruitment

Lymph node and muscle cells were plated in a U-bottom 96-well plate and centrifuged at 500 g for 2 min at 4°C. Cells were stained with 100 μL of Live/Dead aqua dye (BioLegend, Catalog: 423101) and incubated for 20 min at 4°C in the dark. After centrifuging at 500 g for 2 min, the supernatant was removed. Cells were resuspended in Fc block (Anti-CD16, Clone:93, BD, Catalog: 6266549) in PBS with 1% BSA for 20 min, followed by staining with surface antibodies: CD3 (Clone: 17A2, Invitrogen, Catalog: 17-0032-82), Ly6G (Clone: 1A8), BD, Catalog: 7046845), CD11c (Clone: HL3, BD, Catalog: 6209870), F4/80 (Clone: BM8, eBiosciences, Catalog: 15-4801-82), CD103 (Clone: 2E7, BioLegend, Catalog: 121406), NK1.1 (Clone: DX5, BD Biosciences, Catalog: 551114), and MHCII (Clone: M5/114, eBiosciences, Catalog: 4289686) for 1 h at room temperature in the dark. Excess antibodies were washed off three times with 1% BSA in PBS, and the cells were filtered through fluorescence-activated cell sorting tubes. Flow cytometry was performed on an LSRFortessa (BD), and data analysis was conducted using FlowJo. FMO controls were used for surface staining analysis (Figure S9).

Multiplex cytokine analysis

Cytokines from THP-1 supernatants were assessed using a custom 10-spot U-Plex human kit from Meso Scale Discovery (MSD). The concentrations of nine analytes (IFN-α2α, IFN-β, IFN-**γ**, CXCL10, CCL2, CCL3, CCL4, CCL7, and CCL8) were assessed. Cytokines from serum were assessed using a custom 10-spot U-Plex mouse kit from Meso Scale Discovery (MSD). The concentrations of nine analytes (IFN-α, IFN-β, IFN-**γ**, IL-5, IL-6, CCL2, CXCL1, CXCL10, and tumor necrosis factor [TNF]) were assessed.

Statistical analysis

All graphs and statistical analysis were performed in GraphPad Prism V9.3.0 (GraphPad Software, San Diego, CA). Heatmap correlations were determined by Pearson's rank analysis. Statistical significance was defined as a p value <0.05 by one-way analysis of variance (ANOVA) or linear regression.

DATA AVAILABILITY

Data are available on request.

ACKNOWLEDGMENTS

The authors thank KS for assistance in the *in vivo* studies, Ziyin Wang for help on flow panels, and Lesley Rawlinson for lab management. For the purpose of open access, the author has applied a Creative Commons Attribution (CC BY) license to any Author Accepted Manuscript version arising. This study was funded by an EPSRC DTP iCASE award to J.T.. J.T. and R.S. are supported by an NIHR BRC award to Imperial.

AUTHOR CONTRIBUTIONS

H.J.S., conceptualization, investigation, and writing—original draft; B.T.C., conceptualization and resources; D.J.P., investigation, writing—review & editing; A.M.H., supervision and writing—review & editing; R.J.S., supervision and writing—review & editing; R. C., conceptualization, funding acquisition, and writing—review & editing; J.S.T., conceptualization, supervision, funding acquisition, and writing—review & editing.

DECLARATION OF INTERESTS

B.T.C., A.M.H., and R.C. are employees and have an equity interest in Aqdot (Cambridge, UK).

SUPPLEMENTAL INFORMATION

Supplemental information can be found online at https://doi.org/10.1016/j.omtn.2025.102585.

REFERENCES

1. Røttingen, J.-A., Gouglas, D., Feinberg, M., Plotkin, S., Raghavan, K.V., Witty, A., Draghia-Akli, R., Stoffels, P., and Piot, P. (2017). New Vaccines against Epidemic Infectious Diseases. N. Engl. J. Med. Overseas. Ed. *376*, 610–613. https://doi.org/10.1056/nejmp1613577.
2. Ulmer, J.B., Mansoura, M.K., and Geall, A.J. (2015). Vaccines 'on demand': science fiction or a future reality. Expet Opin. Drug Discov. *10*, 101–106. https://doi.org/10.1517/17460441.2015.996128.
3. Pardi, N., Parkhouse, K., Kirkpatrick, E., McMahon, M., Zost, S.J., Mui, B.L., Tam, Y. K., Karikó, K., Barbosa, C.J., Madden, T.D., et al. (2018). Nucleoside-modified mRNA immunization elicits influenza virus hemagglutinin stalk-specific antibodies. Nat. Commun. *9*, 3361. https://doi.org/10.1038/s41467-018-05482-0.
4. McKay, P.F., Hu, K., Blakney, A.K., Samnuan, K., Brown, J.C., Penn, R., Zhou, J., Bouton, C.R., Rogers, P., Polra, K., et al. (2020). Self-amplifying RNA SARS-CoV-2

lipid nanoparticle vaccine candidate induces high neutralizing antibody titers in mice. Nat. Commun. *11*, 3523. https://doi.org/10.1038/s41467-020-17409-9.

5. Baden, L.R., El Sahly, H.M., Essink, B., Kotloff, K., Frey, S., Novak, R., Diemert, D., Spector, S.A., Rouphael, N., Creech, C.B., et al. (2021). Efficacy and Safety of the mRNA-1273 SARS-CoV-2 Vaccine. N. Engl. J. Med. *384*, 403–416. https://doi.org/10.1056/NEJMoa2035389.

6. Polack, F.P., Thomas, S.J., Kitchin, N., Absalon, J., Gurtman, A., Lockhart, S., Perez, J.L., Pérez Marc, G., Moreira, E.D., Zerbini, C., et al. (2020). Safety and Efficacy of the BNT162b2 mRNA Covid-19 Vaccine. N. Engl. J. Med. *383*, 2603–2615. https://doi.org/10.1056/NEJMoa2034577.

7. Sung, Y.K., and Kim, S.W. (2019). Recent advances in the development of gene delivery systems. Biomater. Res. *23*, 8. https://doi.org/10.1186/s40824-019-0156-z.

8. Mendes, B.B., Conniot, J., Avital, A., Yao, D., Jiang, X., Zhou, X., Sharf-Pauker, N., Xiao, Y., Adir, O., Liang, H., et al. (2022). Nanodelivery of nucleic acids. Nat. Rev. Methods Primers *2*, 24. https://doi.org/10.1038/s43586-022-00104-y.

9. Peacocke, E.F., Heupink, L.F., Frønsdal, K., Dahl, E.H., and Chola, L. (2021). Global access to COVID-19 vaccines: a scoping review of factors that may influence equitable access for low and middle-income countries. BMJ Open *11*, e049505. https://doi.org/10.1136/bmjopen-2021-049505.

10. Mislick, K.A., and Baldeschwieler, J.D. (1996). Evidence for the role of proteoglycans in cation-mediated gene transfer. Proc. Natl. Acad. Sci. USA *93*, 12349–12354. https://doi.org/10.1073/pnas.93.22.12349.

11. Phillips, H.R., Tolstyka, Z.P., Hall, B.C., Hexum, J.K., Hackett, P.B., and Reineke, T.M. (2019). Glycopolycation-DNA Polyplex Formulation N/P Ratio Affects Stability, Hemocompatibility, and in Vivo Biodistribution. Biomacromolecules *20*, 1530–1544. https://doi.org/10.1021/acs.biomac.8b01704.

12. Kunath, K., von Harpe, A., Fischer, D., Petersen, H., Bickel, U., Voigt, K., and Kissel, T. (2003). Low-molecular-weight polyethylenimine as a non-viral vector for DNA delivery: comparison of physicochemical properties, transfection efficiency and in vivo distribution with high-molecular-weight polyethylenimine. J. Contr. Release *89*, 113–125. https://doi.org/10.1016/s0168-3659(03)00076-2.

13. Freeman, W.A., Mock, W.L., and Shih, N.Y. (1981). Cucurbituril. J. Am. Chem. Soc. *103*, 7367–7368. https://doi.org/10.1021/ja00414a070.

14. Barrow, S.J., Kasera, S., Rowland, M.J., del Barrio, J., and Scherman, O.A. (2015). Cucurbituril-Based Molecular Recognition. Chem. Rev. *115*, 12320–12406. https://doi.org/10.1021/acs.chemrev.5b00341.

15. Barooah, N., Mohanty, J., and Bhasikuttan, A.C. (2022). Cucurbituril-Based Supramolecular Assemblies: Prospective on Drug Delivery, Sensing, Separation, and Catalytic Applications. Langmuir *38*, 6249–6264. https://doi.org/10.1021/acs.langmuir.2c00556.

16. Das, D., Assaf, K.I., and Nau, W.M. (2019). Applications of Cucurbiturils in Medicinal Chemistry and Chemical Biology. Front. Chem. *7*, 619. https://doi.org/10.3389/fchem.2019.00619.

17. Nikolova, V., Dobrev, S., Kircheva, N., Yordanova, V., Dudev, T., and Angelova, S. (2023). Host-guest complexation of cucurbit[7]uril and cucurbit[8]uril with the antimuscarinic drugs tropicamide and atropine. J. Mol. Graph. Model. *119*, 108380. https://doi.org/10.1016/j.jmgm.2022.108380.

18. Chernikova, E.Y., and Berdnikova, D.V. (2020). Cucurbiturils in nucleic acids research. Chem. Commun. *56*, 15360–15376. https://doi.org/10.1039/d0cc06583h.

19. Huang, Q., Li, S., Ding, Y.-F., Yin, H., Wang, L.-H., and Wang, R. (2018). Macrocycle-wrapped polyethylenimine for gene delivery with reduced cytotoxicity. Biomater. Sci. *6*, 1031–1039. https://doi.org/10.1039/C8BM00022K.

20. Zhang, W., Zhang, H.-Y., Zhang, Y.-H., and Liu, Y. (2015). Fluorescent supramolecular polypseudorotaxane architectures with Ru(ii)/tri(bipyridine) centers as multifunctional DNA reagents. Chem. Commun. *51*, 16127–16130. https://doi.org/10.1039/C5CC07216F.

21. Blakney, A.K., Zhu, Y., McKay, P.F., Bouton, C.R., Yeow, J., Tang, J., Hu, K., Samnuan, K., Grigsby, C.L., Shattock, R.J., and Stevens, M.M. (2020). Big Is Beautiful: Enhanced saRNA Delivery and Immunogenicity by a Higher Molecular Weight, Bioreducible, Cationic Polymer. ACS Nano *14*, 5711–5727. https://doi.org/10.1021/acsnano.0c00326.

22. Dong, R., Zhou, L., Wu, J., Tu, C., Su, Y., Zhu, B., Gu, H., Yan, D., and Zhu, X. (2011). A supramolecular approach to the preparation of charge-tunable dendritic polycations for efficient gene delivery. Chem. Commun. *47*, 5473–5475. https://doi.org/10.1039/C1CC10934K.

23. Boeckle, S., von Gersdorff, K., van der Piepen, S., Culmsee, C., Wagner, E., and Ogris, M. (2004). Purification of polyethylenimine polyplexes highlights the role of free polycations in gene transfer. J. Gene Med. *6*, 1102–1111. https://doi.org/10.1002/jgm.598.

24. Yue, Y., Jin, F., Deng, R., Cai, J., Dai, Z., Lin, M.C.M., Kung, H.F., Mattebjerg, M.A., Andresen, T.L., and Wu, C. (2011). Revisit complexation between DNA and polyethylenimine–effect of length of free polycationic chains on gene transfection. J. Contr. Release *152*, 143–151. https://doi.org/10.1016/j.jconrel.2011.03.020.

25. Dai, Z., Gjetting, T., Mattebjerg, M.A., Wu, C., and Andresen, T.L. (2011). Elucidating the interplay between DNA-condensing and free polycations in gene transfection through a mechanistic study of linear and branched PEI. Biomaterials *32*, 8626–8634. https://doi.org/10.1016/j.biomaterials.2011.07.044.

26. Pezzoli, D., Giupponi, E., Mantovani, D., and Candiani, G. (2017). Size matters for in vitro gene delivery: investigating the relationships among complexation protocol, transfection medium, size and sedimentation. Sci. Rep. *7*, 44134. https://doi.org/10.1038/srep44134.

27. Aranda-Barradas, M.E., Trejo-López, S.E., Real, A.D., Álvarez-Almazán, S., Méndez-Albores, A., García-Tovar, C.G., González-Díaz, F.R., and Miranda-Castro, S.P. (2022). Effect of molecular weight of chitosan on the physicochemical, morphological, and biological properties of polyplex nanoparticles intended for gene delivery. Carbohydrate Polymer Technologies and Applications *4*, 100228. https://doi.org/10.1016/j.carpta.2022.100228.

28. Haladjova, E., Panseri, S., Montesi, M., Rossi, A., Skandalis, A., Pispas, S., and Rangelov, S. (2023). Influence of DNA Type on the Physicochemical and Biological Properties of Polyplexes Based on Star Polymers Bearing Different Amino Functionalities. Polymers (Basel) *15*, 894. https://doi.org/10.3390/polym15040894.

29. Fischer, D., Li, Y., Ahlemeyer, B., Krieglstein, J., and Kissel, T. (2003). In vitro cytotoxicity testing of polycations: influence of polymer structure on cell viability and hemolysis. Biomaterials *24*, 1121–1131. https://doi.org/10.1016/S0142-9612(02)00445-3.

30. Fischer, D., Bieber, T., Li, Y., Elsässer, H.-P., and Kissel, T. (1999). A Novel Non-Viral Vector for DNA Delivery Based on Low Molecular Weight, Branched Polyethylenimine: Effect of Molecular Weight on Transfection Efficiency and Cytotoxicity. Pharm. Res. *16*, 1273–1279. https://doi.org/10.1023/A:1014861900478.

31. Cheng, Y., Sellers, D.L., Tan, J.-K.Y., Peeler, D.J., Horner, P.J., and Pun, S.H. (2017). Development of switchable polymers to address the dilemma of stability and cargo release in polycationic nucleic acid carriers. Biomaterials *127*, 89–96. https://doi.org/10.1016/j.biomaterials.2017.02.036.

32. Blakney, A.K., McKay, P.F., Hu, K., Samnuan, K., Jain, N., Brown, A., Thomas, A., Rogers, P., Polra, K., Sallah, H., et al. (2021). Polymeric and lipid nanoparticles for delivery of self-amplifying RNA vaccines. J. Contr. Release *338*, 201–210. https://doi.org/10.1016/j.jconrel.2021.08.029.

33. Tregoning, J.S., Stirling, D.C., Wang, Z., Flight, K.E., Brown, J.C., Blakney, A.K., McKay, P.F., Cunliffe, R.F., Murugaiah, V., Fox, C.B., et al. (2023). Formulation, inflammation, and RNA sensing impact the immunogenicity of self-amplifying RNA vaccines. Mol. Ther. Nucleic Acids *31*, 29–42. https://doi.org/10.1016/j.omtn.2022.11.024.

34. Neeland, M.R., Shi, W., Collignon, C., Meeusen, E.N.T., Didierlaurent, A.M., and de Veer, M.J. (2018). The adjuvant system AS01 up-regulates neutrophil CD14 expression and neutrophil-associated antigen transport in the local lymphatic network. Clin. Exp. Immunol. *192*, 46–53. https://doi.org/10.1111/cei.13088.

35. Bakke, S.S., Aune, M.H., Niyonzima, N., Pilely, K., Ryan, L., Skjelland, M., Garred, P., Aukrust, P., Halvorsen, B., Latz, E., et al. (2017). Cyclodextrin Reduces Cholesterol Crystal-Induced Inflammation by Modulating Complement Activation. J. Immunol. *199*, 2910–2920. https://doi.org/10.4049/jimmunol.1700302.

36. Pashkina, E., Aktanova, A., Boeva, O., Bykova, M., Gavrilova, E., Goiman, E., Kovalenko, E., Saleh, N., Grishina, L., and Kozlov, V. (2024). Evaluation of the Immunosafety of Cucurbit[n]uril In Vivo. Pharmaceutics *16*, 127. https://doi.org/10.3390/pharmaceutics16010127.

37. (2015). The Diffusion Barrier Technique, Practical Aspects and Data interpretation. https://www.materials-talks.com/wp-content/uploads/2015/09/MRK1651-02-The-Diffusion-Barrier-Technique-Practical-Aspects-and-Data-interpretation.pdf.

OMTN, Volume *36*

Supplemental information

Manipulating the delivery and immunogenicity of DNA vaccines through the addition of CB[8] to cationic polymers

Hadijatou J. Sallah, Benjamin T. Cheesman, David J. Peeler, Andrew M. Howe, Robin J. Shattock, Roger Coulston, and John S. Tregoning

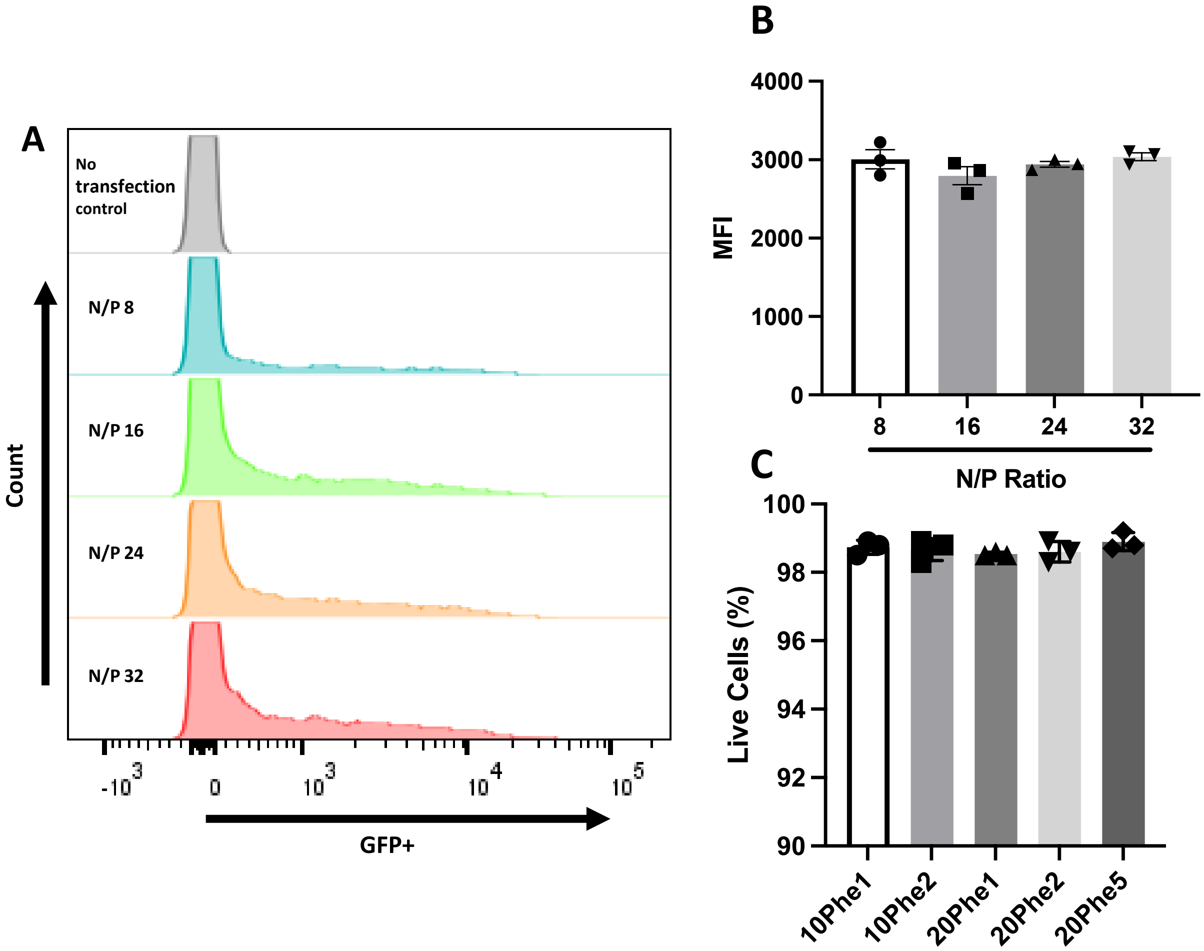

Figure S1. Flow cytometry histograms of live HEK293T following transfection with 20Phe1+CB[8] GFP pDNA formulations at different N/P ratios cells (A), mean fluorescence intensity (MFI) (B) and and cell viability as determined by live/Dead staining (C).

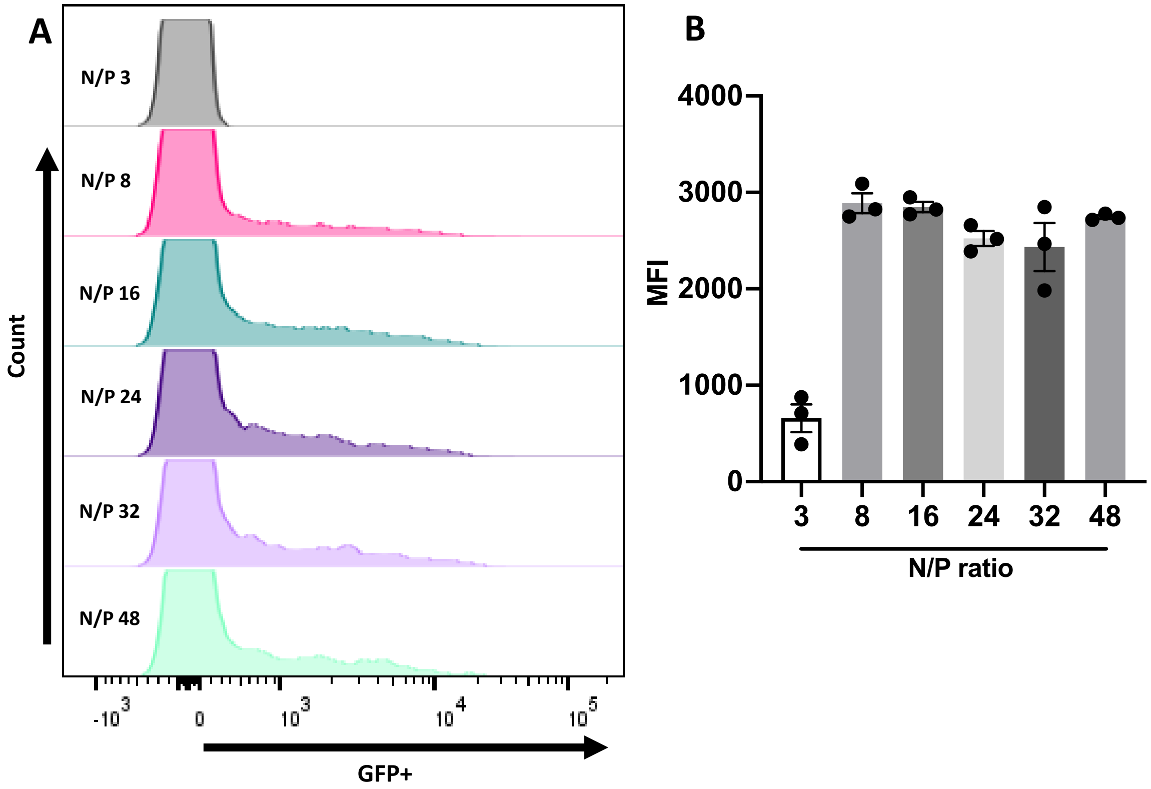

Figure S2. Flow cytometry histograms of live HEK293T following transfection with 20Phe1+CB[8] GFP pDNA formulations at different N/P ratios (A) and mean fluorescence intensity (MFI) (B)

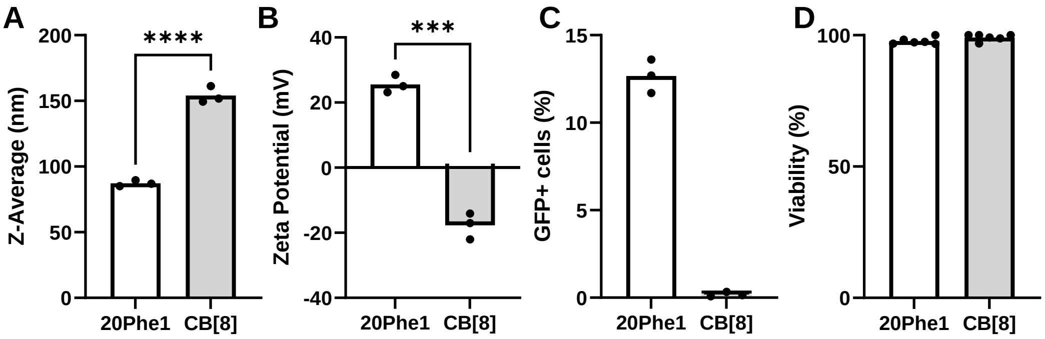

Figure S3. Characterisation of individual polyplex components. Particle diameter (A) and zeta potential (B) of polyplexes formed from CB[8] or 20Phe1 only at N/P ratio of 24:1. Quantification of GFP expression by cell percentage (%) 24 h post-transfection (C). Cell viability of cells following transfection (D).

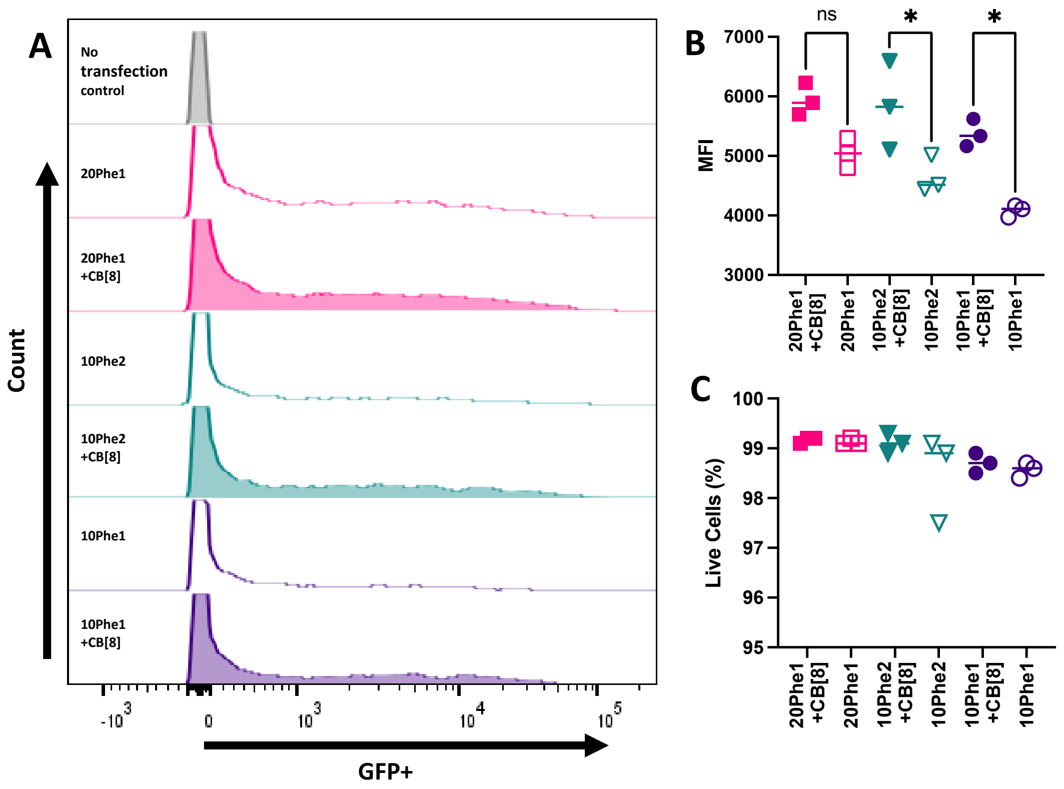

Figure S4. Flow cytometry histograms of live HEK293T following transfection with 20Phe1, 10Phe2 and 10Phe1 ± CB[8] GFP pDNA formulations at different N/P ratios cells (A), mean fluorescence intensity (MFI) (B) and cell viability as determined by live/Dead staining (C) * $p < 0.05$.

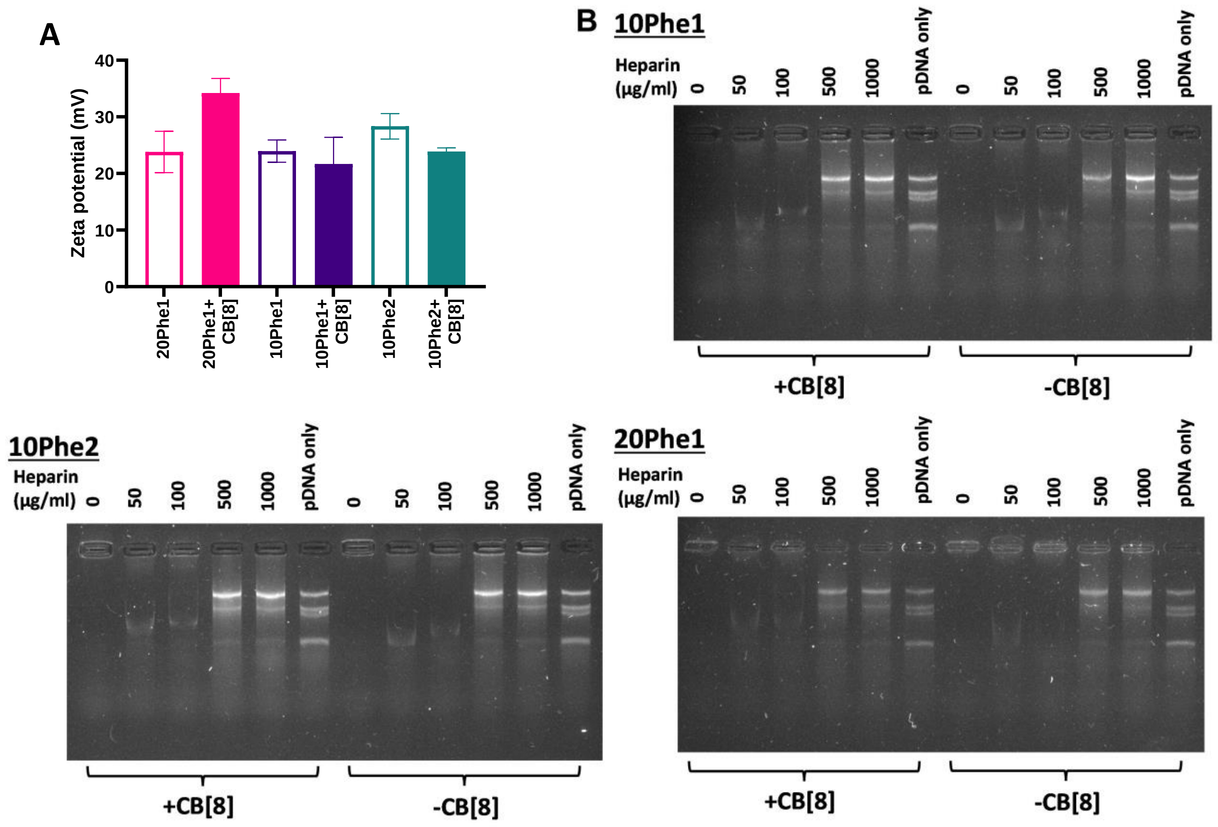

Figure S5. Particle charges and unpackaging of polymer formulations with and without CB[8]. Zeta potential of polyplexes formed using the CB[8] polymer system and pDNA at N/P 32 with or without CB[8] (A).

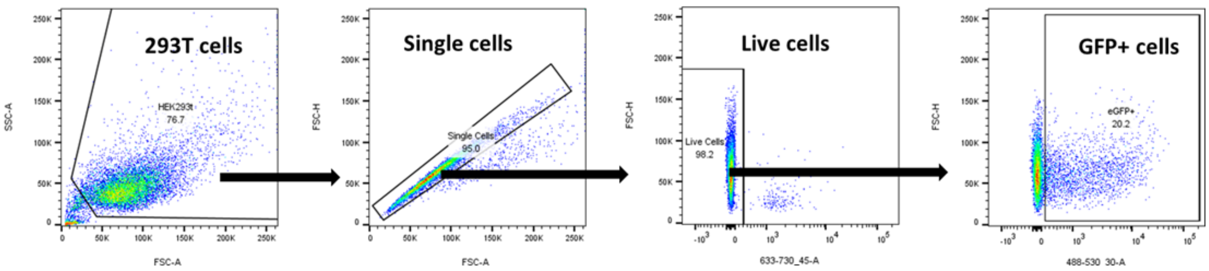

Figure S6. GFP gating strategy. HEK293T/17 cells harvested following transfection and gene expression assessed by flow cytometry assay. Samples were analysed according to gating strategy shown.

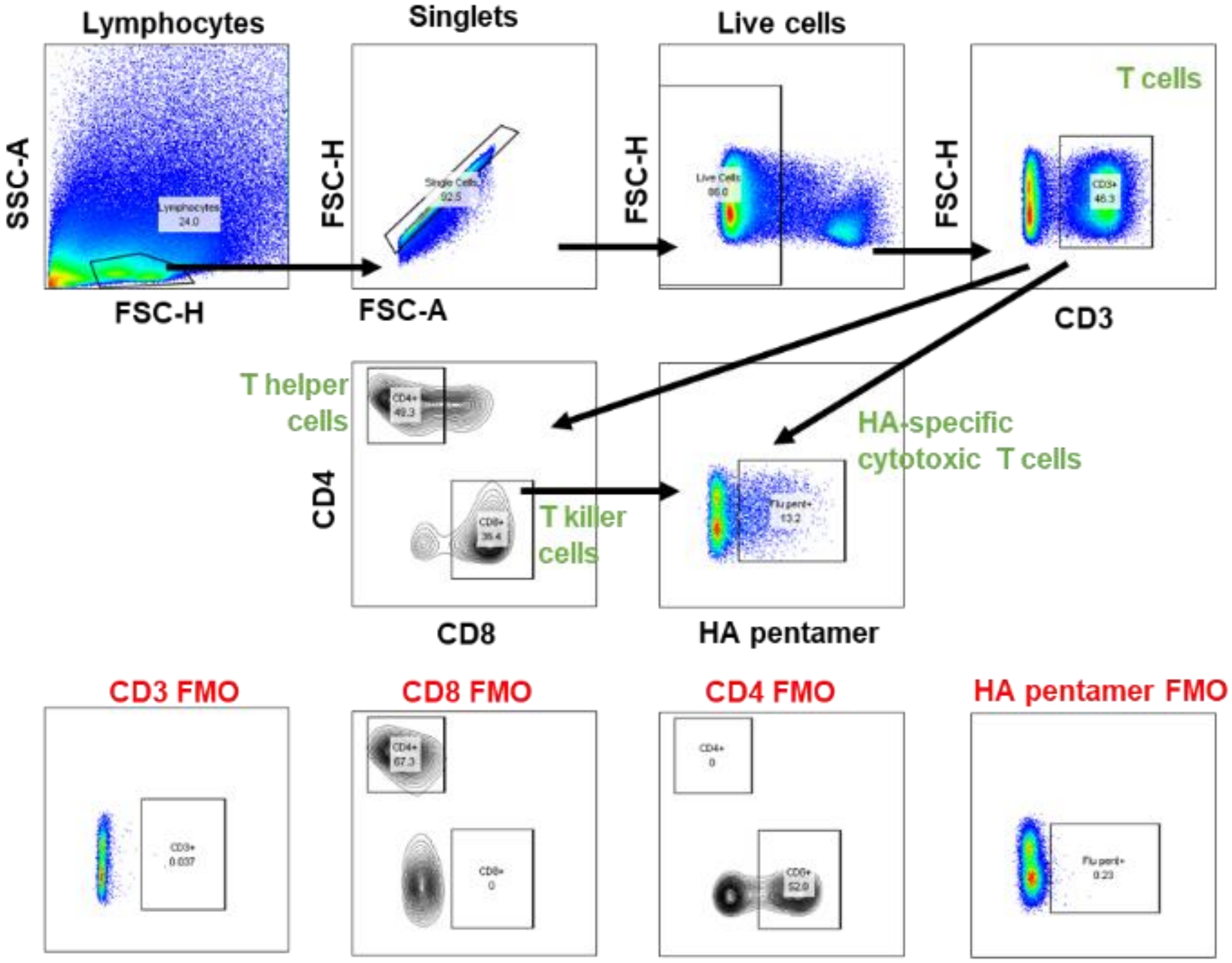

Figure S7. T cell phenotyping gating strategy. Cells were collected from lung prior to flow cytometry assay. Samples were analysed according to gating strategy shown.

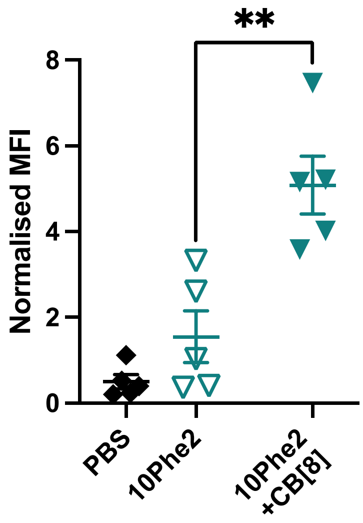

Figure S8. Normalised mean fluorescence intensity (MFI) of live GFP+ cells following administration of 10Phe2 GFP pDNA formulations ± CB[8]. ** $p < 0.005$.

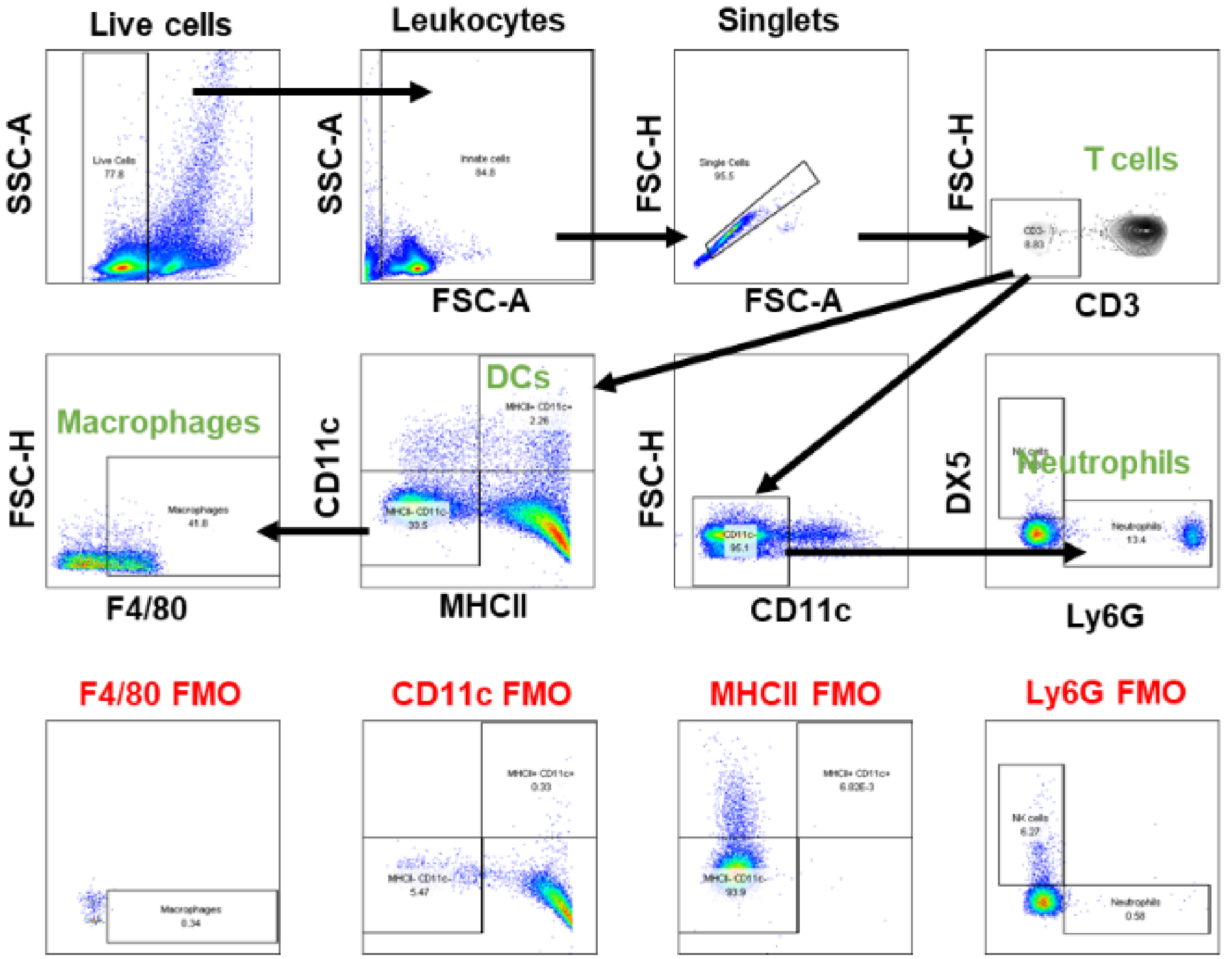

Figure S9. Innate cell recruitment gating strategy. Cells were collected from lymph node or muscle tissue prior to flow cytometry assay. Samples were analysed according to gating strategy shown.
